# Associations of body size and morphology with cardiometabolic health in children: the contribution of genetic factors

**DOI:** 10.1002/oby.24196

**Published:** 2024-12-05

**Authors:** Karri Silventoinen, José Maia, Reijo Sund, Élvio R. Gouveia, António Antunes, Gonçalo Marques, Martine Thomis, Aline Jelenkovic, Jaakko Kaprio, Duarte Freitas

**Affiliations:** ^1^ Helsinki Institute for Demography and Population Health, University of Helsinki Helsinki Finland; ^2^ Centre of Research, Education, Innovation, and Intervention in Sport (CIFI2D), Faculty of Sport University of Porto Porto Portugal; ^3^ Institute of Clinical Medicine, University of Eastern Finland Kuopio Finland; ^4^ Department of Physical Education and Sport University of Madeira Funchal Portugal; ^5^ LARSYS, Interactive Technologies Institute Funchal Portugal; ^6^ Physical Activity, Sports & Health Research Group Department of Movement Sciences, Faculty of Movement and Rehabilitation Sciences, KU Leuven Leuven Belgium; ^7^ Department of Genetics, Physical Anthropology and Animal Physiology, Faculty of Science and Technology University of the Basque Country (UPV/EHU) Bilbao Spain; ^8^ Institute for Molecular Medicine Finland (FIMM), HiLIFE University of Helsinki Helsinki Finland

## Abstract

**Objective:**

We analyzed how anthropometric measures predict cardiometabolic health and how genetic and environmental factors contribute to these associations.

**Methods:**

Data on 8 indicators of cardiometabolic health, 21 anthropometric measures, and 11 anthropometric indices were available for 216 twin pairs of individuals age 3 to 18 years living in the Autonomous Region of Madeira, Portugal (51% girls). Genetic twin modeling was used to estimate genetic and environmental correlations between the cardiometabolic and anthropometric indicators.

**Results:**

Anthropometric indicators were positively associated with blood pressure and triglycerides and inversely associated with high‐density lipoprotein cholesterol. The associations with glucose, low‐density lipoprotein cholesterol, total cholesterol, and heart rate were close to zero. BMI and waist circumference showed similar or slightly higher absolute correlations with cardiometabolic health indicators compared with other anthropometric indices. Additive genetic and unique environmental correlations were at the same level as trait correlations.

**Conclusions:**

BMI and waist circumference provide information on cardiometabolic health that is not less accurate than that provided by more comprehensive anthropometric indices. These associations reflect causal associations between obesity and cardiometabolic disorders rather than only shared genetic associations. Measuring obesity is important for monitoring cardiometabolic risks and can be accomplished using simple indicators at the population level.

**Study Importance**

**What is already known?**

- BMI is associated with cardiometabolic health indicators, but it is still unclear whether more detailed anthropometric measures could improve the risk estimates.

**What does this study add?**

- BMI showed equal or even stronger associations with key metabolic health indicators than other anthropometric traits, and both genetic and environmental factors contributed to these associations.

**How might these results change the direction of research or the focus of clinical practice?**

- BMI offers an effective tool at the population level to measure obesity‐related risks of cardiometabolic health.

## INTRODUCTION

Childhood is an important phase of life for establishing adult cardiometabolic health, and factors such as childhood adiposity, hypertension, and hyperlipidemia predict the risk of cardiovascular diseases (CVD) in adulthood [1]. In particular, adiposity has been linked to other cardiometabolic risk factors in children [2] and can significantly increase the risk of CVD due to its strong association with obesity in adulthood [3]. Childhood obesity has increased dramatically worldwide during the last decades and creates a major public health challenge [4]. However, previous studies have also consistently shown that short stature is associated with an increased risk of CVD in various populations [5], suggesting that anthropometric traits other than obesity indicators can also predict cardiometabolic health. This highlights the significance of anthropometric measures in public health research to identify children at high risk for CVD in adulthood.

Studies of relationships between anthropometry and cardiometabolic health have mostly collected information on a limited number of anthropometric measures, typically height and weight, and occasionally on waist and hip circumferences [6]. However, these traits may be insufficient to fully capture the CVD risk associated with body size, morphology, and composition. First, not all children defined as having obesity develop cardiometabolic complications, leading to the hypothesis that the accumulation of adipose tissue in different parts of the body has different metabolic consequences [2]. It has been suggested that waist circumference (WC) is a better predictor of cardiometabolic diseases than body mass index (BMI) [7], but it is still largely an open question whether more detailed anthropometric measures could improve the predictive power. Second, it has been speculated that environmental factors affect the growth of different body parts differently. Leg length has been suggested to be more susceptible to the influence of environmental stressors and a better predictor of CVD risk than trunk length [8], although this hypothesis has also been challenged [9]. Modern methods such as dual‐energy x‐ray absorptiometry, computer tomography, and magnetic resonance imaging can provide detailed information on the amount and distribution of muscle and adipose tissue in the body [10], but they are too expensive for large‐scale epidemiological studies and health screenings aimed at the general population. Therefore, it would be beneficial for public health purposes to identify a set of anthropometric traits that best capture cardiometabolic risks in children and adolescents.

When examining the connections between anthropometric traits and cardiometabolic health indicators, genetics warrant special attention. Genetic factors explain a significant proportion of the variation in both anthropometric [11] and cardiometabolic traits [12]. Twin [13] and genome‐wide association studies [14] have demonstrated that largely the same genetic factors account for BMI variation from mid‐childhood to adulthood. Thus, genetic factors may also contribute to the association between cardiometabolic factors during childhood and the risk of developing CVD in adulthood in addition to environmental factors. The shared genetic background of anthropometric and cardiometabolic traits can be due to the direct influence of genes on different traits (i.e., pleiotropy), causal associations among the traits, or the influence of common background factors. If anthropometric and cardiometabolic traits share environmental influences, this may reflect causal influences in either or both directions or permit the identification of exposures that affect both. Diet or physical activity, which are known to affect both cardiometabolic health and body composition [15], could contribute to these exposures.

In this study, we aim to analyze the associations between anthropometric traits and cardiometabolic health indicators in children and adolescents. Additionally, we will use our genetically informative twin design to analyze the shared genetic and environmental factors underlying these associations. We will use data containing information on 21 anthropometric measures, 11 indices calculated from these anthropometric measures, and eight cardiometabolic health indicators, allowing for a comprehensive analysis of the associations between anthropometry and cardiometabolic health. Our research questions are as follows: 1) Which anthropometric traits are most closely associated with the indicators of cardiometabolic health?; 2) Can the predictive power be increased by using indices that merge information from several anthropometric measures?; and 3) To what extent do anthropometric and cardiometabolic traits share the same genetic and environmental background?

## METHODS

The data were obtained from the Madeira Twin Family Study, which was conducted in the Autonomous Region of Madeira, Portugal, in 2007 and 2008 after the Scientific Board of the University of Madeira had approved the study protocol [16]. First, the executive boards of all public and private schools in Madeira were contacted to inquire whether they had twin students (Figure S1). Second, an invitation letter was sent to all 434 families with twin children to participate in this study. Third, the twin children from all 216 families who agreed to participate were invited to a clinical examination in the capital city of Funchal. The participating twins and/or their parents/legal guardians provided written informed consent. The age range of the participants was 3 to 18 years, with 51% being girls. During the examination, the children provided a blood sample after fasting for a minimum of 8 h. Zygosity was determined using 15 autosomal, unlinked loci and a sex‐determining marker [17]. From the twin pairs, 87 were monozygotic (MZ), 73 were same‐sex dizygotic (DZ), and 56 were opposite‐sex DZ pairs.

Glucose, high‐density lipoprotein (HDL) cholesterol, low‐density lipoprotein (LDL) cholesterol, triglycerides, and total cholesterol were measured from plasma (Cobas Integra 800, Roche Diagnostics). Systolic and diastolic blood pressure (SBP and DBP, respectively) and heart rate were measured twice in a seated position on the nondominant arm after 5 to 10 min of rest. An automatic blood pressure monitor (Model Omron HEM‐780 N3, OMRON Healthcare) with cuff sizes appropriate for the child's age and weight was used. The means of two measures were recorded. If the difference in SBP or DBP was more than 5 mm Hg, blood pressure measures were repeated. Anthropometric traits were measured based on a standardized protocol [18]. All measurements were done while participants were wearing a swimsuit, without shoes and with jewelry removed. All one‐sided measurements were taken from the left side of the body. Together, we had the following 21 anthropometric measures: height; leg length (calculated by subtracting sitting height from height); weight; four body diameters (i.e., biacromial, bicristal, humerus, and femur); seven body circumferences (i.e., waist, hip, calf, thigh, upper arm, forearm, and upper arm flexed); and seven skinfold thickness measures (i.e., triceps, biceps, subscapular, suprailiac, calf, front thigh, and abdominal). The details of these measures, including the measurement devices used, have been described elsewhere [19]. Based on these measures, several indices were calculated. BMI was calculated by dividing weight in kilograms by height in meters squared. Waist to height ratio (WHtR) was calculated by dividing WC by height, and waist to hip ratio (WHR) was calculated by dividing WC by hip circumference. Percentage fat, fat mass (FM), and fat‐free mass (FFM) were estimated from the triceps and subscapular skinfolds using the equations of Slaughter et al. [20]. The three somatotype components were calculated using the Heath and Carter [21] method, i.e., endomorphy (relative fatness), mesomorphy (relative musculoskeletal development), and ectomorphy (relative linearity). Finally, we conducted a factor analysis using the varimax rotation for all anthropometric measures, as described in detail elsewhere [19]. Two uncorrelated factors were extracted: Factor 1 we interpreted to reflect body fatness; and Factor 2 we interpreted to reflect body tallness/robustness. We calculated the *z* scores of height and BMI using the 2007 World Health Organization (WHO) reference charts [22]. For other anthropometric measures and cardiometabolic traits, we calculated the *z* scores by adjusting them for age and age squared separately in boys and girls. Furthermore, we checked the distributions of all traits and standardized the distributions showing skewness (waist, hip, and thigh circumferences; abdominal, calf, biceps, subscapular, suprailiac, and triceps skinfolds; triglycerides; and endomorphy) by the logarithmic transformation.

The data were analyzed using genetic twin modeling based on different genetic similarities of MZ and DZ twins: whereas MZ twins are virtually genetically identical at the gene sequence level, DZ twins share half of their genetic variation, which is similar to ordinary siblings [23]. Based on this principle, it is possible to decompose trait variance into genetic and environmental components. Additive genetic variance (A; correlation of 1 within MZ and 0.5 within DZ pairs) includes the effects of all loci affecting the trait. Shared environmental variance (C; correlation of 1 within both MZ and DZ twins) includes the effects of all environmental factors making co‐twins similar. Unique environmental variance (E; correlation of 0 within both MZ and DZ twins) includes the effects of all environmental factors making co‐twins dissimilar, including measurement error. We have previously shown that the assumptions of twin modeling (i.e., the same means and standard deviations [SD] for MZ and DZ twins, as well as for the first and second co‐twin within a pair) were not violated, and the best model was the additive genetic/unique environmental (AE) model for the cardiometabolic health indicators [24], the anthropometric measures [19], and the somatotype traits [25] in these data. These previous studies have also reported the estimates of additive genetic and unique environmental relative variances for these traits, as well as all descriptive statistics. We found that the estimates of additive genetic and unique environmental relative variance components were roughly similar for younger children (i.e., less than age 12 years, mainly at the prepubertal stage) and older children (i.e., age 12 years or older, mainly at the puberty or post‐puberty stage) without showing any systematic age differences [24]. Therefore, in this study, we pooled all children together and presented these estimates for the whole cohort because we had not done this previously. These estimates also offer important information for the reader to evaluate the importance of genetic and environmental correlations. The corresponding estimates for the anthropometric measures [19] and the somatotype traits [25] have been reported previously.

Our main analyses involved estimating the additive genetic and unique environmental correlations between the anthropometric and cardiometabolic traits using bivariate Cholesky decomposition. Cholesky decomposition is a model‐free method that decomposes all variation and covariation in the data into uncorrelated latent factors [26]. Using this approach, we decomposed the covariation between anthropometric and cardiometabolic traits into genetic and environmental covariances and then standardized them to provide additive genetic and unique environmental correlations. The genetic twin modeling was conducted using the OpenMx package version 3.0.2 (The R Project for Statistical Computing) [27]. This package uses the maximum likelihood estimator to estimate parameter values and their confidence intervals (CI) through the structural equations modeling framework.

Finally, we analyzed how the anthropometric traits were associated with metabolic abnormalities by calculating the area under the receiver operating characteristic curve (AUC) with 95% CI. We fitted each anthropometric trait into the logistic model, including age, age squared, and sex, and analyzed how much the AUC differed among the traits. Our participants generally had a good metabolic profile; therefore, we used borderline limits for metabolic abnormalities [28]. For blood glucose, only five participants had levels meeting the hyperglycemia definition for children (i.e., ≥100 mg/dL) [29]; therefore, we did not analyze this further. The AUC estimates and all descriptive statistics were calculated using Stata/MP for Windows software version 18.0 (StataCorp LLC), with the cluster option to correct the standard errors for the effect of intrapair correlations [30].

## RESULTS

Table 1 presents the descriptive statistics for cardiometabolic traits stratified by sex. The glucose level was slightly higher in boys, and the levels of triglycerides and heart rate were higher in girls. Otherwise, we did not find sex differences in the cardiometabolic traits. The proportions of borderline metabolic abnormalities varied from 7% for low HDL cholesterol in boys to 34% for hypercholesterolemia in girls, without statistically significant sex differences (Table 2). Next, we calculated the relative variance components of additive genetic and unique environmental factors for cardiometabolic traits in the pooled data of boys and girls (Table 3). Additive genetic factors explained around 60% (i.e., SBP and DBP) to over 80% (i.e., total, HDL, and LDL cholesterol) of the variation in these traits. When we performed these analyses stratified by sex (Table S1), we found no systematic sex differences in the magnitude of additive genetic and unique environmental factors, and the 95% CI overlapped for boys and girls.

**TABLE 1:** Descriptive statistics of cardiometabolic traits by sex.

	Boys		Girls		*p* value of sex difference^a^
	Mean	SD	Mean	SD	
SBP, mm Hg	108	11	107	10	0.444
DBP, mm Hg	62	8	62	8	0.359
Glucose, mg/dL	83	8	81	7	0.016
Total cholesterol, mg/dL	156	27	158	29	0.550
HDL cholesterol, mg/dL	60	14	60	15	0.617
LDL cholesterol, mg/dL	81	22	83	25	0.445
Triglycerides, mg/dL	63	29	69	28	0.045
Heart rate, beats/min	78	13	85	14	<0.0001

Abbreviations: DBP, diastolic blood pressure; HDL, high‐density lipoprotein; LDL, low‐density lipoprotein; SBP, systolic blood pressure.

^a^ Adjusted for age.

**TABLE 2:** Prevalence of borderline metabolic abnormalities with 95% CI by sex.^a^

	Boys			Girls			*p* value of sex difference^b^
	%	95% CI		%	95% CI		
		LL	UL		LL	UL	
Hypercholesterolemia	29	22	37	34	27	42	0.335
Low HDL cholesterol	7	4	12	10	7	15	0.389
High LDL cholesterol	12	8	18	16	12	22	0.330
High triglycerides	24	18	32	32	26	41	0.078
Hypertension	17	12	24	16	12	22	0.780

Abbreviations: HDL, high‐density lipoprotein; LL, lower limit; LDL, low‐density lipoprotein; UL, upper limit.

^a^ Limits used for the borderline metabolic abnormalities: ≥170 mg/dL for total cholesterol, ≥110 mg/dL for LDL cholesterol, ≤40 mg/dL for HDL cholesterol, ≥75 mg/dL in children younger than age 10 years and ≥90 mg/dL for children age 10 years and older for triglycerides, the highest decile of age‐adjusted systolic or diastolic blood pressure.

^b^ Adjusted for age.

**TABLE 3:** Proportion of variation of cardiometabolic traits explained by additive genetic and unique environmental factors in the pooled data of boys and girls.

	Additive genetic factors			Unique environmental factors		
	a^2^	95% CI		e^2^	95% CI	
		LL	UL		LL	UL
SBP	0.57	0.43	0.68	0.43	0.32	0.57
DBP	0.61	0.48	0.72	0.39	0.28	0.52
Glucose	0.66	0.55	0.74	0.34	0.26	0.45
Total cholesterol	0.84	0.77	0.88	0.16	0.12	0.23
HDL cholesterol	0.86	0.81	0.90	0.14	0.10	0.19
LDL cholesterol	0.84	0.78	0.88	0.16	0.12	0.22
Triglycerides	0.71	0.61	0.78	0.29	0.22	0.39
Heart rate	0.70	0.60	0.78	0.30	0.22	0.40

Abbreviations: a^2^, additive genetic variation; DBP, diastolic blood pressure; e^2^, unique environmental variation; HDL, high‐density lipoprotein; LL, lower limit; LDL, low‐density lipoprotein; SBP, systolic blood pressure; UL, upper limit.

**FIGURE 1:**
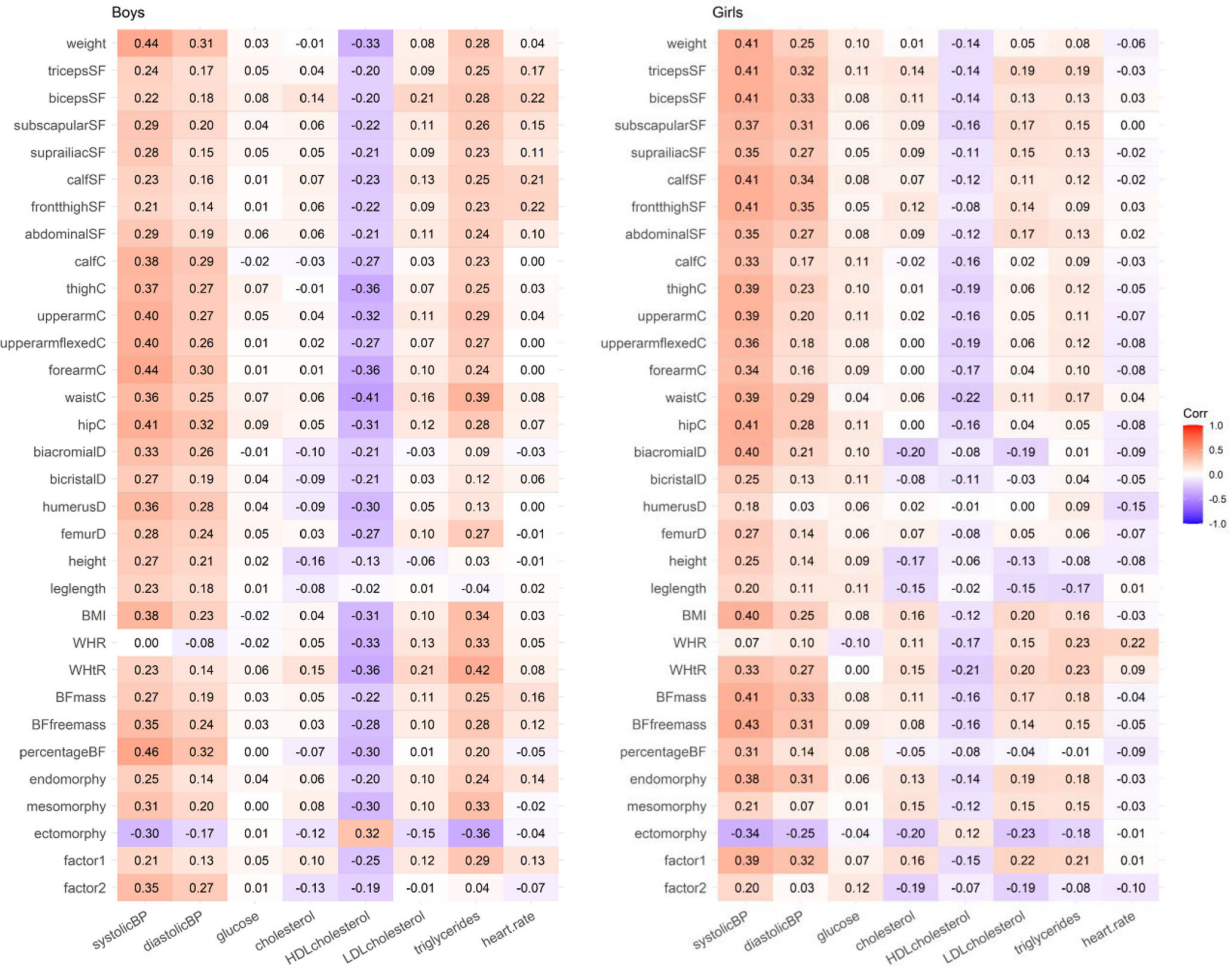
Trait correlations between anthropometric and cardiometabolic traits in boys and girls. BF, body fat; BP, blood pressure; C, circumference; D, diameter; SF, skinfold; WHR, waist to hip ratio; WtHR, waist to height ratio. [Color figure can be viewed at wileyonlinelibrary.com]

After the univariate models, we analyzed the associations between anthropometric and cardiometabolic traits. Figure 1 presents the trait correlations between these traits in boys and girls (95% CI are available in Table S2). The correlations were generally highest for SBP, whereas somewhat lower correlations were found for DBP, HDL cholesterol, and triglycerides. Greater anthropometric measures indicating larger body size were associated with worse cardiometabolic health (i.e., higher levels of blood pressure and triglycerides and lower levels of HDL cholesterol). For glucose, total cholesterol, LDL cholesterol, and heart rate, most of the correlations were close to zero. In general, BMI and WC had similar or slightly higher absolute values of correlations with blood pressure, HDL cholesterol, and triglycerides than the other anthropometric measures such as the skinfold thicknesses, WHR, and WHtR. Additionally, most of the correlations were higher for height compared with leg length. The absolute correlations for more complex indices, i.e., the three measures of FM and FFM, the three somatotype measures, and the two factors reflecting body fatness (Factor 1) and body tallness/robustness (Factor 2), were not generally higher when compared with BMI and WC.

We did not find major sex differences in the size of these correlations (the statistics of sex differences are presented in Table S3). Only 29 of these 256 correlations showed statistically significant sex differences when using a conventional level of statistical significance (*p* < 0.05), and none of them were significant when using the Bonferroni‐corrected significance level (*p* < 0.0002). However, this general similarity of correlations may conceal some real sex differences. For triglycerides, the correlations were systematically higher in boys, and a similar sex difference existed for the correlations between skinfold thicknesses and heart rate, showing moderate and mainly statistically significant correlations in boys but virtually zero correlations in girls (Table S2).

**FIGURE 2:**
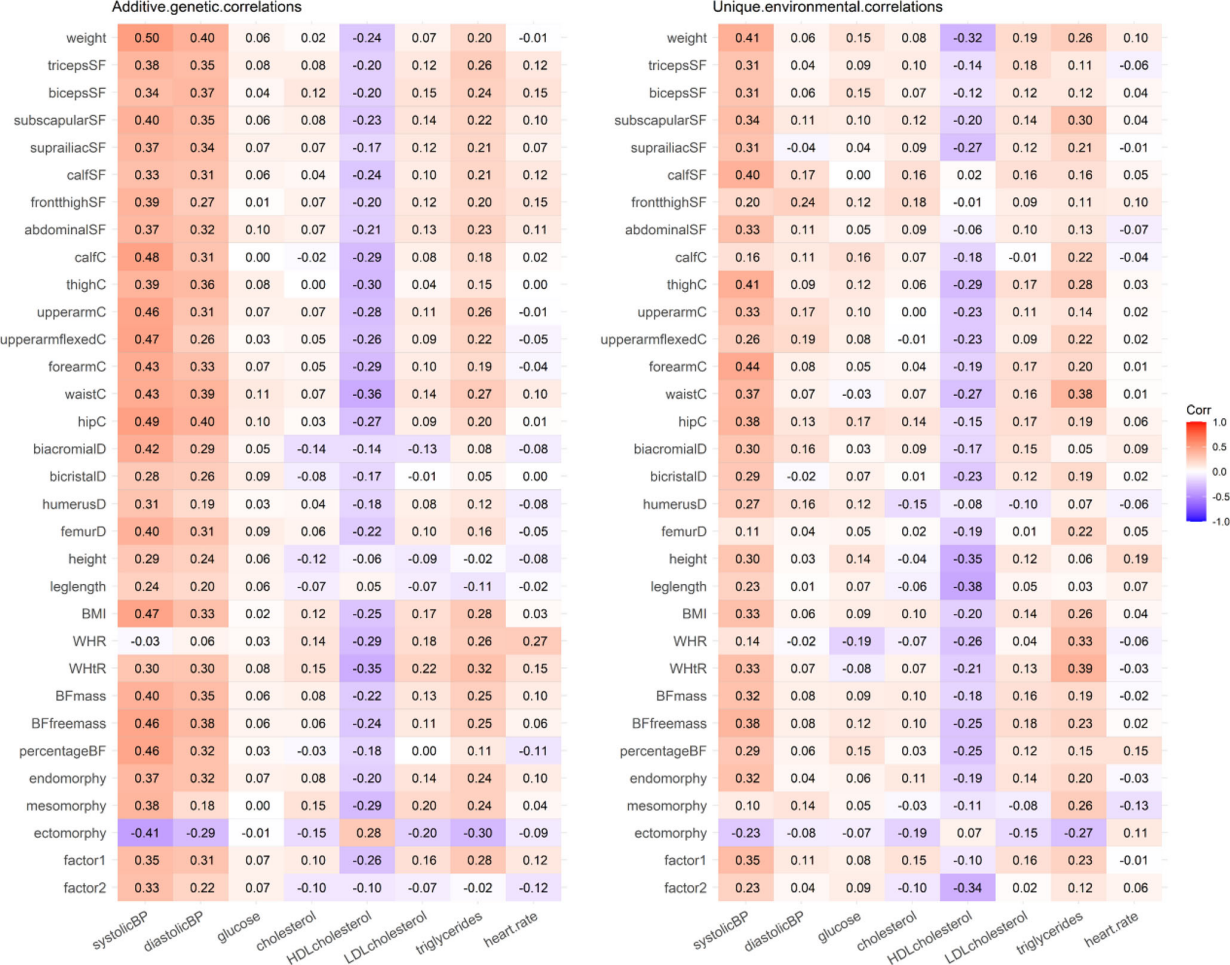
Additive genetic and unique environmental correlations between anthropometric and cardiometabolic traits in the pooled data of boys and girls. BF, body fat; BP, blood pressure; C, circumference; D, diameter; SF, skinfold; WHR, waist to hip ratio; WtHR, waist to height ratio. [Color figure can be viewed at wileyonlinelibrary.com]

We then decomposed these trait correlations into additive genetic and unique environmental correlations as presented in Figure 2, using pooled data from boys and girls (95% CI are presented in Table S4). Additive genetic and unique environmental correlations were generally at similar levels and close to the trait correlations. The exceptions were the correlations of DBP, for which additive genetic correlations were moderate and statistically significant, whereas most of the unique environmental correlations were close to zero. When we conducted sex‐stratified analyses, no major sex differences were found for additive genetic (Table S5) or unique environmental correlations (Table S6). However, in these sex‐specific analyses, additive genetic correlations were moderate and mostly statistically significant for skinfold thicknesses and heart rate in boys, whereas, for girls, they were close to zero.

Finally, we analyzed how the anthropometric measures predict borderline metabolic abnormalities (Table S7). Some differences were found in the AUC estimates among the anthropometric measures, but they were relatively small (i.e., differences of 0.16 or less). Furthermore, the 95% CI overlapped among most of the models.

## DISCUSSION

This study comprehensively analyzes the associations between anthropometry and cardiometabolic health in children using 21 anthropometric measures, 11 anthropometric indices, and eight cardiometabolic indicators. Earlier studies exploring these associations have focused on obesity indicators [2]. Our findings indicate that obesity indicators are associated with higher blood pressure and triglycerides, as well as lower HDL cholesterol. However, the associations for the other risk factors of CVD (i.e., glucose, LDL cholesterol, and heart rate) were weaker or virtually nonexistent. BMI is the most widely used indicator of obesity, but it has been criticized because it does not discriminate between FFM and FM and does not take into account body fat distribution, which also has metabolic consequences [31]. However, our results indicate that BMI has similar or even slightly stronger correlations with the cardiometabolic health indicators compared with other obesity indices such as WC, WHR, and WHtR or other anthropometric measures such as different skinfold thicknesses and body circumferences, which are suggested to provide better information on adiposity and its distribution than BMI [6]. Therefore, our findings suggest that BMI can be considered a reliable obesity indicator at the population level.

In addition to obesity, previous studies have examined the associations between height and cardiometabolic health. Research has shown that short adult stature is associated with a higher risk of CVD in many populations, which may indicate childhood undernutrition as a risk factor for CVD [5]. However, the association between shorter adult stature and higher CVD mortality has also been found within MZ twin pairs [32], suggesting that prenatal conditions may also contribute to this association. MZ twins are likely to share their postnatal environment, but their intrauterine environment can differ considerably, especially in monochorionic pregnancies [33]. Our study revealed that height and leg length were positively associated with SBP and DBP. Additionally, they were inversely associated with total cholesterol, but this was partly due to HDL cholesterol. Therefore, we cannot conclude that taller children present better cardiometabolic health. Additionally, we found no evidence to support the hypothesis that leg length is more strongly associated with cardiometabolic health than height. It is worth noting that a positive association between height and blood pressure has been systematically found in children [34], whereas, in adults, most studies have reported inverse associations between these traits [35]. It is unclear whether these conflicting results suggest a biological and developmental difference in the association between blood pressure and height in children versus adults or whether socioeconomic factors may explain this changing association because they are positively associated with stature and inversely associated with the risk of CVD [36]. The third explanation for these differences is that they reflect cohort differences. Owing to an improved standard of living, height differences among individuals may no longer be associated with differences in undernutrition, as they were in previous generations in industrialized countries and still are in many developing countries [37].

In addition to single anthropometric measures and simple indices, we included indices based on several anthropometric measures assessing body composition and morphology. However, our results did not indicate that they would provide more accurate information on cardiometabolic health than the simpler indicators. Somatotype is a commonly used indicator of human physique and body shape. We found that both endomorphy (relative fatness) and mesomorphy (relative musculoskeletal development) were associated with worse cardiometabolic health, whereas ectomorphy (relative linearity) was associated with better cardiometabolic health. These results were consistent with a previous Spanish study of 429 nuclear families, which also found that endomorphy and mesomorphy were associated with higher blood pressure and ectomorphy was associated with lower blood pressure [38]. However, in this Spanish study, the correlations were lower than in our study, which may be due to a much wider age range (i.e., 4–61 years) in the two‐generation study. The estimates of both FM and FFM were associated with worse cardiometabolic health when using the equations of Slaughter et al. [20]. This may indicate that this method cannot correctly discriminate between FM and FFM. We also extracted two uncorrelated factors indicating relative fatness and body tallness/robustness, which were associated with worse cardiometabolic health. Thus, ectomorphy was the only indicator associated with better cardiometabolic health. However, the absolute values of the correlations for all these indices were lower or only slightly higher than those for BMI.

Because we had information on MZ and DZ twins, we were able to decompose the trait correlations found into additive genetic and unique environmental correlations. For most of the anthropometric traits that showed correlations with cardiometabolic indicators, the additive genetic and unique environmental correlations were at the same level. This supports the hypothesis that causality contributes to these associations. For example, obesity can lead to cardiometabolic health disorders, as supported by results from randomized controlled trials [39]. If these correlations were only caused by pleiotropic effects, we would expect the unique environmental correlations to be lower than the genetic correlations. However, we discovered two noteworthy exceptions. First, we observed that DBP had stronger genetic correlations than environmental correlations with the anthropometric traits. Second, we found that skinfold thicknesses showed genetic correlations with heart rate in boys, but this was not found in girls. Both DBP [40] and heart rate [41] have been considered as risk factors for CVD, despite their somewhat lower predictive power compared with SBP and blood lipids. Our results may suggest that there are environmental factors that affect DBP and heart rate, but that they are not related to cardiometabolic health, which may attenuate the associations found at the phenotypic level.

Our study has certain strengths and limitations. It is the most detailed study of anthropometric and cardiometabolic traits to date, to the best of our knowledge. In addition to 21 anthropometric measures, we had several indicators of cardiometabolic health, including not only blood pressure and lipid measures but also glucose and heart rate. This enabled us to thoroughly examine how anthropometry is associated with various aspects of cardiometabolic health. However, we missed some important cardiometabolic indicators, including fasting insulin, insulin after the oral glucose tolerance test, and C‐reactive protein. Furthermore, our twin data allowed us to analyze the influence of genetic and environmental factors on these correlations. Genome‐wide association studies provide an alternative method to estimate genetic correlations. However, they require a larger sample size, which is not feasible when using detailed anthropometric measures. Additionally, polygenic risk scores are not yet available for most of the anthropometric traits used in this study. Our main limitation is that our sample size was not large enough to study potential changes in these correlations over childhood and adolescence. In our previous study, we found only minor and unsystematic differences in the role of genetic and environmental factors on cardiometabolic traits in our data between younger and older children [24]. However, it is possible that the associations between anthropometric and cardiometabolic measures may change throughout childhood. Also, we did not have measures based on dual‐energy x‐ray absorptiometry or other accurate methods allowing us to directly measure percentage fat, FM, FFM, and their distribution in the body. It is probable that these measures would be more closely linked to cardiometabolic factors than the anthropometric measures available in our study. However, these methods are not feasible in large‐scale epidemiological studies and health check‐ups aimed at the general population due to their high costs. Our measurement protocol was not optimal for the components of stature, as we needed to estimate leg length by subtracting sitting height from stature. This may have increased uncertainty in the assessment of leg length.

Our research population was metabolically healthy, and around 30% met the criteria for borderline hypercholesterolemia in children. For the other metabolic disturbances, this proportion was smaller. Twin pregnancies differ from singleton pregnancies and often result in lower birth weight and other characteristics in newborns [42], but the anthropometric differences between twins and singletons mostly disappear during the first 3 years of life [43]. Furthermore, in adulthood, no differences are observed in CVD risk between twins and singletons [44]. Even though we do not have reasons to believe that the correlations between anthropometric and metabolic measures would be substantially different in other populations, replicating these correlations in other groups with different cardiometabolic risk profiles would be warranted and could help to generalize the results.

## CONCLUSION

Anthropometric measures provide information on cardiometabolic health, but simple indicators such as BMI and WC did not perform worse than more comprehensive measures. Genetic and environmental factors showed similar correlations, suggesting that these associations also reflect causal relationships at the phenotypic level rather than only pleiotropic effects. Anthropometric measures are important in assessing the increased risk of cardiometabolic diseases associated with adiposity. At the population level, this does not require comprehensive body measurements, but simple indicators can provide sufficient information to identify children who may be prone to developing cardiometabolic diseases in the future. However, at the individual level, a more detailed screening of body composition is needed to identify children whose higher body weight is not due to adiposity but rather to higher muscle mass.

## FUNDING INFORMATION

The project “Genetic and environmental influences on physical activity, fitness and health: the Madeira family study” was supported by the Fundação para a Ciência e a Tecnologia (FCT; the Portuguese National Funding Agency for Science, Research and Technology; reference POCI/DES/56834/2004). Karri Silventoinen and Jaakko Kaprio have been supported by the European Union's Horizon Europe Research and Innovation program under grant agreement number 101080117. Views and opinions expressed are however those of the author(s) only and do not necessarily reflect those of the European Union. Neither the European Union nor the granting authority can be held responsible for them. Jaakko Kaprio has been supported by the Academy of Finland Center of Excellence in Complex Disease Genetics (grant #352792). Aline Jelenkovic has been supported by the Evolutionary Biology and Human Health consolidated research group (IT1693‐22, Basque Government).

## CONFLICT OF INTEREST STATEMENT

The authors declared no conflicts of interest.

## Supporting information


**Data S1.** Supporting Information.
