# Supplementary material for: Associations of body size and morphology with cardiometabolic health in children: the contribution of genetic factors
Source: Obesity (Silver Spring). 2024 Dec 5;33(1):125–33. doi: 10.1002/oby.24196 (PMC11664301; doi:10.1002/oby.24196)
Supplement: Supplementary file 1 — Data S1. Supporting Information. [file OBY-33-125-s001.pdf]

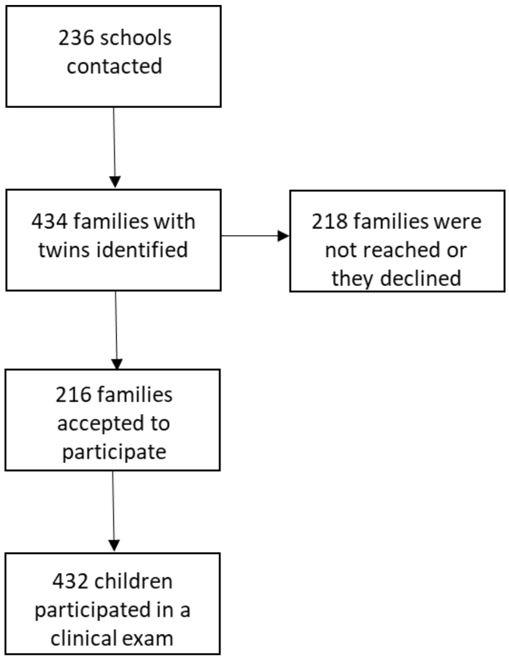

Supplementary figure S1. Flow chart of data collection.

Supplementary table S1. The proportion of variation of cardiometabolic traits explained by additive genetic and unique environmental factors in boys and girls.

	Boys						Girls					
	Additive genetic factors			Unique environmental factors			Additive genetic factors			Unique environmental factors		
	a^2	95% CI		e^2	95% CI		a^2	95% CI		e^2	95% CI	
		LL	UL		LL	UL		LL	UL		LL	UL
SBP	0.68	0.49	0.80	0.32	0.20	0.51	0.47	0.24	0.64	0.53	0.36	0.76
DBP	0.64	0.42	0.77	0.36	0.23	0.58	0.59	0.38	0.74	0.41	0.26	0.62
Glucose	0.60	0.42	0.73	0.40	0.27	0.58	0.75	0.62	0.84	0.25	0.16	0.38
Total cholesterol	0.82	0.71	0.89	0.18	0.11	0.29	0.85	0.75	0.91	0.15	0.09	0.25
HDL cholesterol	0.89	0.82	0.93	0.11	0.07	0.18	0.84	0.75	0.90	0.16	0.10	0.25
LDL cholesterol	0.81	0.71	0.87	0.19	0.13	0.29	0.86	0.77	0.91	0.14	0.09	0.23
Triglycerides	0.68	0.51	0.79	0.32	0.21	0.49	0.74	0.60	0.83	0.26	0.17	0.40
Heart rate	0.75	0.60	0.84	0.25	0.16	0.40	0.67	0.51	0.78	0.33	0.22	0.49

Abbreviations: SBP=systolic blood pressure, DBP=diastolic blood pressure, HDL=high-density lipoprotein, LDL=low-density lipoprotein

Supplementary table S2. Trait correlations between anthropometric and metabolic measures with 95% confidence intervals in boys and girls.

	Boys								Girls							
	A	B	C	D	E	F	G	H	A	B	C	D	E	F	G	H
1	0.44<br>0.32,<br>0.54	0.31<br>0.19,<br>0.43	0.03<br>-0.11,<br>0.16	-0.01<br>-0.15,<br>0.13	-0.33<br>-0.45,<br>-0.20	0.08<br>-0.05,<br>0.22	0.28<br>0.15,<br>0.40	0.04<br>-0.09,<br>0.18	0.41<br>0.30,<br>0.52	0.25<br>0.12,<br>0.37	0.10<br>-0.03,<br>0.23	0.01<br>-0.12,<br>0.15	-0.14<br>-0.27,<br>-0.01	0.05<br>-0.08,<br>0.18	0.08<br>-0.06,<br>0.21	-0.06<br>-0.19,<br>0.08
2	0.24<br>0.11,<br>0.37	0.17<br>0.03,<br>0.30	0.05<br>-0.09,<br>0.18	0.04<br>-0.10,<br>0.18	-0.20<br>-0.33,<br>-0.07	0.09<br>-0.04,<br>0.23	0.25<br>0.11,<br>0.37	0.17<br>0.04,<br>0.30	0.41<br>0.29,<br>0.51	0.32<br>0.19,<br>0.43	0.11<br>-0.03,<br>0.24	0.14<br>0.00,<br>0.27	-0.14<br>-0.27,<br>-0.01	0.19<br>0.06,<br>0.31	0.19<br>0.05,<br>0.31	-0.03<br>-0.16,<br>0.10
3	0.22<br>0.08,<br>0.34	0.18<br>0.04,<br>0.31	0.08<br>-0.06,<br>0.21	0.14<br>0.01,<br>0.28	-0.20<br>-0.33,<br>-0.07	0.21<br>0.07,<br>0.34	0.28<br>0.15,<br>0.40	0.22<br>0.08,<br>0.34	0.41<br>0.29,<br>0.51	0.33<br>0.21,<br>0.45	0.08<br>-0.05,<br>0.21	0.11<br>-0.03,<br>0.24	-0.14<br>-0.26,<br>-0.00	0.13<br>-0.01,<br>0.26	0.13<br>-0.01,<br>0.26	0.03<br>-0.10,<br>0.16
4	0.29<br>0.15,<br>0.41	0.20<br>0.06,<br>0.32	0.04<br>-0.09,<br>0.18	0.06<br>-0.07,<br>0.20	-0.22<br>-0.35,<br>-0.09	0.11<br>-0.03,<br>0.25	0.26<br>0.12,<br>0.38	0.15<br>0.01,<br>0.28	0.37<br>0.25,<br>0.48	0.31<br>0.18,<br>0.42	0.06<br>-0.07,<br>0.19	0.09<br>-0.04,<br>0.22	-0.16<br>-0.29,<br>-0.03	0.17<br>0.04,<br>0.29	0.15<br>0.01,<br>0.28	0.00<br>-0.13,<br>0.13
5	0.28<br>0.15,<br>0.40	0.15<br>0.02,<br>0.29	0.05<br>-0.09,<br>0.18	0.05<br>-0.09,<br>0.19	-0.21<br>-0.33,<br>-0.07	0.09<br>-0.04,<br>0.23	0.23<br>0.10,<br>0.36	0.11<br>-0.03,<br>0.24	0.35<br>0.22,<br>0.46	0.27<br>0.14,<br>0.39	0.05<br>-0.08,<br>0.18	0.09<br>-0.05,<br>0.22	-0.11<br>-0.24,<br>0.02	0.15<br>0.02,<br>0.28	0.13<br>-0.00,<br>0.26	-0.02<br>-0.15,<br>0.11
6	0.23<br>0.10,<br>0.36	0.16<br>0.02,<br>0.29	0.01<br>-0.13,<br>0.15	0.07<br>-0.07,<br>0.20	-0.23<br>-0.36,<br>-0.10	0.13<br>-0.01,<br>0.26	0.25<br>0.11,<br>0.37	0.21<br>0.08,<br>0.34	0.41<br>0.30,<br>0.52	0.34<br>0.22,<br>0.45	0.08<br>-0.05,<br>0.21	0.07<br>-0.06,<br>0.20	-0.12<br>-0.25,<br>0.01	0.11<br>-0.02,<br>0.24	0.12<br>-0.02,<br>0.25	-0.02<br>-0.15,<br>0.11
7	0.21<br>0.07,<br>0.33	0.14<br>0.00,<br>0.27	0.01<br>-0.12,<br>0.15	0.06<br>-0.08,<br>0.19	-0.22<br>-0.34,<br>-0.08	0.09<br>-0.05,<br>0.22	0.23<br>0.10,<br>0.36	0.22<br>0.09,<br>0.35	0.41<br>0.29,<br>0.51	0.35<br>0.22,<br>0.46	0.05<br>-0.08,<br>0.18	0.12<br>-0.02,<br>0.24	-0.08<br>-0.21,<br>0.05	0.14<br>0.01,<br>0.27	0.09<br>-0.05,<br>0.22	0.03<br>-0.11,<br>0.16
8	0.29<br>0.15,<br>0.41	0.19<br>0.05,<br>0.31	0.06<br>-0.08,<br>0.20	0.06<br>-0.07,<br>0.20	-0.21<br>-0.34,<br>-0.08	0.11<br>-0.03,<br>0.24	0.24<br>0.11,<br>0.37	0.10<br>-0.04,<br>0.23	0.35<br>0.23,<br>0.46	0.27<br>0.15,<br>0.39	0.08<br>-0.05,<br>0.21	0.09<br>-0.04,<br>0.22	-0.12<br>-0.25,<br>0.01	0.17<br>0.03,<br>0.29	0.13<br>-0.01,<br>0.26	0.02<br>-0.11,<br>0.15
9	0.38<br>0.26,<br>0.49	0.29<br>0.16,<br>0.41	-0.02<br>-0.16,<br>0.12	-0.03<br>-0.17,<br>0.11	-0.27<br>-0.39,<br>-0.14	0.03<br>-0.10,<br>0.17	0.23<br>0.10,<br>0.36	0.00<br>-0.14,<br>0.13	0.33<br>0.20,<br>0.44	0.17<br>0.04,<br>0.30	0.11<br>-0.02,<br>0.24	-0.02<br>-0.15,<br>0.12	-0.16<br>-0.29,<br>-0.03	0.02<br>-0.11,<br>0.16	0.09<br>-0.04,<br>0.23	-0.03<br>-0.16,<br>0.10
10	0.37<br>0.24,<br>0.48	0.27<br>0.14,<br>0.40	0.07<br>-0.07,<br>0.21	-0.01<br>-0.15,<br>0.13	-0.36<br>-0.48,<br>-0.24	0.07<br>-0.07,<br>0.21	0.25<br>0.11,<br>0.37	0.03<br>-0.10,<br>0.17	0.39<br>0.27,<br>0.50	0.23<br>0.10,<br>0.35	0.10<br>-0.03,<br>0.23	0.01<br>-0.12,<br>0.15	-0.19<br>-0.31,<br>-0.06	0.06<br>-0.07,<br>0.19	0.12<br>-0.01,<br>0.25	-0.05<br>-0.19,<br>0.08
11	0.40<br>0.27,<br>0.51	0.27<br>0.14,<br>0.39	0.05<br>-0.09,<br>0.18	0.04<br>-0.10,<br>0.18	-0.32<br>-0.44,<br>-0.20	0.11<br>-0.03,<br>0.25	0.29<br>0.16,<br>0.42	0.04<br>-0.10,<br>0.17	0.39<br>0.27,<br>0.49	0.20<br>0.07,<br>0.33	0.11<br>-0.02,<br>0.24	0.02<br>-0.12,<br>0.15	-0.16<br>0.29,<br>-0.03	0.05<br>-0.08,<br>0.18	0.11<br>-0.02,<br>0.24	-0.07<br>-0.20,<br>0.06

12	0.40<br>0.27,<br>0.51	0.26<br>0.12,<br>0.38	0.01<br>-0.13,<br>0.15	0.02<br>-0.12,<br>0.15	-0.27<br>-0.39,<br>-0.14	0.07<br>-0.07,<br>0.21	0.27<br>0.14,<br>0.39	0.00<br>-0.14,<br>0.13	0.36<br>0.23,<br>0.47	0.18<br>0.05,<br>0.30	0.08<br>-0.05,<br>0.21	-0.00<br>-0.14,<br>0.13	-0.19<br>-0.31,<br>-0.06	0.06<br>-0.07,<br>0.19	0.12<br>-0.01,<br>0.25	-0.08<br>-0.21,<br>0.05
13	0.44<br>0.32,<br>0.54	0.30<br>0.17,<br>0.42	0.01<br>-0.13,<br>0.15	0.01<br>-0.13,<br>0.15	-0.36<br>-0.47,<br>-0.23	0.10<br>-0.03,<br>0.24	0.24<br>0.10,<br>0.37	0.00<br>-0.14,<br>0.13	0.34<br>0.22,<br>0.45	0.16<br>0.03,<br>0.29	0.09<br>-0.04,<br>0.23	0.00<br>-0.14,<br>0.13	-0.17<br>-0.29,<br>-0.03	0.04<br>-0.09,<br>0.17	0.10<br>-0.04,<br>0.23	-0.08<br>-0.21,<br>0.06
14	0.36<br>0.23,<br>0.47	0.25<br>0.12,<br>0.37	0.07<br>-0.07,<br>0.20	0.06<br>-0.08,<br>0.20	-0.41<br>-0.52,<br>-0.29	0.16<br>0.02,<br>0.29	0.39<br>0.26,<br>0.50	0.08<br>-0.05,<br>0.22	0.39<br>0.27,<br>0.50	0.29<br>0.17,<br>0.41	0.04<br>-0.09,<br>0.18	0.06<br>-0.08,<br>0.19	-0.22<br>-0.35,<br>-0.09	0.11<br>-0.02,<br>0.24	0.17<br>0.03,<br>0.30	0.04<br>-0.09,<br>0.17
15	0.41<br>0.29,<br>0.52	0.32<br>0.20,<br>0.44	0.09<br>-0.05,<br>0.22	0.05<br>-0.09,<br>0.18	-0.31<br>-0.43,<br>-0.18	0.12<br>-0.02,<br>0.25	0.28<br>0.15,<br>0.40	0.07<br>-0.07,<br>0.21	0.41<br>0.29,<br>0.51	0.28<br>0.15,<br>0.40	0.11<br>-0.03,<br>0.23	0.00<br>-0.13,<br>0.14	-0.16<br>-0.28,<br>-0.02	0.04<br>-0.09,<br>0.17	0.05<br>-0.08,<br>0.19	-0.08<br>-0.21,<br>0.05
16	0.33<br>0.21,<br>0.45	0.26<br>0.13,<br>0.38	-0.01<br>-0.15,<br>0.13	-0.10<br>-0.23,<br>0.04	-0.21<br>-0.33,<br>-0.07	-0.03<br>-0.16,<br>0.11	0.09<br>-0.05,<br>0.23	-0.03<br>-0.16,<br>0.11	0.40<br>0.28,<br>0.50	0.21<br>0.08,<br>0.33	0.10<br>-0.03,<br>0.23	-0.20<br>0.32,<br>-0.07	-0.08<br>-0.21,<br>0.05	-0.19<br>-0.32,<br>-0.06	0.01<br>-0.12,<br>0.15	-0.09<br>-0.22,<br>0.04
17	0.27<br>0.14,<br>0.40	0.19<br>0.06,<br>0.32	0.04<br>-0.10,<br>0.18	-0.09<br>-0.23,<br>0.04	-0.21<br>-0.34,<br>-0.08	0.03<br>-0.10,<br>0.17	0.12<br>-0.02,<br>0.25	0.06<br>-0.08,<br>0.19	0.25<br>0.12,<br>0.37	0.13<br>-0.00,<br>0.26	0.11<br>-0.03,<br>0.24	-0.08<br>-0.21,<br>0.05	-0.11<br>-0.24,<br>0.02	-0.03<br>-0.16,<br>0.10	0.04<br>-0.10,<br>0.17	-0.05<br>-0.18,<br>0.09
18	0.36<br>0.23,<br>0.47	0.28<br>0.15,<br>0.40	0.04<br>-0.10,<br>0.17	-0.09<br>-0.22,<br>0.05	-0.30<br>-0.42,<br>-0.16	0.05<br>-0.09,<br>0.18	0.13<br>-0.01,<br>0.27	0.00<br>-0.13<br>0.14	0.18<br>0.05,<br>0.31	0.03<br>-0.11,<br>0.16	0.06<br>-0.08,<br>0.19	0.02<br>-0.11,<br>0.16	-0.01<br>-0.14,<br>0.13	0.00<br>-0.13,<br>0.14	0.09<br>-0.05,<br>0.22	-0.15<br>-0.28,<br>-0.02
19	0.28<br>0.15,<br>0.40	0.24<br>0.10,<br>0.36	0.05<br>-0.09,<br>0.19	0.03<br>-0.10,<br>0.17	-0.27<br>-0.39,<br>-0.13	0.10<br>-0.04,<br>0.24	0.27<br>0.13,<br>0.39	-0.01<br>-0.15,<br>0.13	0.27<br>0.14,<br>0.39	0.14<br>0.01,<br>0.27	0.06<br>-0.08,<br>0.19	0.07<br>-0.07,<br>0.20	-0.08<br>-0.21,<br>0.05	0.05<br>-0.08,<br>0.18	0.06<br>-0.08,<br>0.20	-0.07<br>-0.20,<br>0.06
20	0.27<br>0.13,<br>0.39	0.21<br>0.08,<br>0.34	0.02<br>-0.11,<br>0.16	-0.16<br>-0.29,<br>-0.02	-0.13<br>-0.26,<br>0.01	-0.06<br>-0.20,<br>0.08	0.03<br>-0.11,<br>0.17	-0.01<br>-0.15,<br>0.12	0.25<br>0.12,<br>0.37	0.14<br>0.01,<br>0.27	0.09<br>-0.05,<br>0.22	-0.17<br>-0.30,<br>-0.04	-0.06<br>-0.19,<br>0.07	-0.13<br>-0.26,<br>0.00	-0.08<br>-0.22,<br>0.05	-0.08<br>-0.21,<br>0.05
21	0.23<br>0.10,<br>0.36	0.18<br>0.04,<br>0.31	0.01<br>-0.13,<br>0.15	-0.08<br>-0.22,<br>0.06	-0.02<br>-0.16,<br>0.12	0.01<br>-0.13,<br>0.15	-0.04<br>-0.18,<br>0.10	0.02<br>-0.12,<br>0.16	0.20<br>0.07,<br>0.32	0.11<br>-0.02,<br>0.24	0.11<br>-0.02,<br>0.24	-0.15<br>-0.28,<br>-0.02)	-0.02<br>-0.15,<br>0.11	-0.15<br>-0.27,<br>-0.01	-0.17<br>-0.30,<br>-0.03	0.01<br>-0.12,<br>0.14
22	0.38<br>0.26,<br>0.50	0.23<br>0.09,<br>0.35	-0.02<br>-0.16,<br>0.12	0.04<br>-0.09,<br>0.18	-0.31<br>-0.43,<br>-0.18	0.10<br>-0.04,<br>0.23	0.34<br>0.21,<br>0.46	0.03<br>-0.10,<br>0.17	0.40<br>0.28,<br>0.51	0.25<br>0.12,<br>0.37	0.08<br>-0.06,<br>0.21	0.16<br>0.02,<br>0.28	-0.12<br>-0.25,<br>0.01	0.20<br>0.06,<br>0.32	0.16<br>0.02,<br>0.28	-0.03<br>-0.16,<br>0.10
23	0.00<br>-0.14,<br>0.14	-0.08<br>-0.22,<br>0.05	-0.02<br>-0.16,<br>0.11	0.05<br>-0.09,<br>0.19	-0.33<br>-0.45,<br>-0.21	0.13<br>-0.01,<br>0.26	0.33<br>0.20,<br>0.45	0.05<br>-0.09,<br>0.18	0.07<br>-0.06,<br>0.20	0.10<br>-0.03,<br>0.23	-0.10<br>-0.23,<br>0.03	0.11<br>-0.03,<br>0.24	-0.17<br>-0.30,<br>-0.04	0.15<br>0.02,<br>0.28	0.23<br>0.10,<br>0.36	0.22<br>0.09,<br>0.35

24	0.23<br>0.09,<br>0.36	0.14<br>-0.00,<br>0.27	0.06<br>-0.08,<br>0.19	0.15<br>0.01,<br>0.28	-0.36<br>-0.48,<br>-0.24	0.21<br>0.08,<br>0.34	0.42<br>0.29,<br>0.53	0.08<br>-0.05,<br>0.22	0.33<br>0.20,<br>0.44	0.27<br>0.15,<br>0.39	-0.00<br>-0.14,<br>0.13	0.15<br>0.02,<br>0.28	-0.21<br>-0.34,<br>-0.08	0.20<br>0.07,<br>0.32	0.23<br>0.10,<br>0.36	0.09<br>-0.05,<br>0.22
25	0.27<br>0.14,<br>0.39	0.19<br>0.05,<br>0.31	0.03<br>-0.11,<br>0.16	0.05<br>-0.08,<br>0.19	-0.22<br>-0.35,<br>-0.09	0.11<br>-0.03,<br>0.24	0.25<br>0.12,<br>0.38	0.16<br>0.02,<br>0.29	0.41<br>0.29,<br>0.51	0.33<br>0.20,<br>0.44	0.08<br>-0.05,<br>0.21	0.11<br>-0.02,<br>0.24	-0.16<br>-0.29,<br>-0.03	0.17<br>0.04,<br>0.30	0.18<br>0.05,<br>0.31	-0.04<br>-0.17,<br>0.09
26	0.35<br>0.22,<br>0.46	0.24<br>0.11,<br>0.37	0.03<br>-0.11,<br>0.17	0.03<br>-0.10,<br>0.17	-0.28<br>-0.40,<br>-0.14	0.10<br>-0.03,<br>0.24	0.28<br>0.14,<br>0.40	0.12<br>-0.01,<br>0.26	0.43<br>0.32,<br>0.53	0.31<br>0.19,<br>0.43	0.09<br>-0.04,<br>0.22	0.08<br>-0.06,<br>0.21	-0.16<br>-0.29,<br>-0.03	0.14<br>0.00,<br>0.26	0.15<br>0.02,<br>0.28	-0.05<br>-0.18,<br>0.08
27	0.46<br>0.34,<br>0.56	0.32<br>0.19,<br>0.43	0.00<br>-0.14,<br>0.14	-0.07<br>-0.21,<br>0.06	-0.30<br>-0.42,<br>-0.16	0.01<br>-0.13,<br>0.15	0.20<br>0.06,<br>0.33	-0.05<br>-0.19,<br>0.08	0.31<br>0.19,<br>0.43	0.14<br>0.01,<br>0.27	0.08<br>-0.05,<br>0.22	-0.05<br>-0.19,<br>0.08	-0.08<br>-0.21,<br>0.06	-0.04<br>-0.18,<br>0.09	-0.01<br>-0.15,<br>0.12	-0.09<br>-0.22,<br>0.05
28	0.25<br>0.11,<br>0.37	0.14<br>0.01,<br>0.27	0.04<br>-0.10,<br>0.17	0.06<br>-0.08,<br>0.20	-0.20<br>-0.33,<br>-0.06	0.10<br>-0.04,<br>0.23	0.24<br>0.10,<br>0.37	0.14<br>0.01,<br>0.27	0.38<br>0.26,<br>0.49	0.31<br>0.19,<br>0.43	0.06<br>-0.07,<br>0.19	0.13<br>0.00,<br>0.26	-0.14<br>-0.27,<br>-0.00	0.19<br>0.06,<br>0.32	0.18<br>0.04,<br>0.31	-0.03<br>-0.16,<br>0.11
29	0.31<br>0.18,<br>0.43	0.20<br>0.07,<br>0.33	0.00<br>-0.14,<br>0.13	0.08<br>-0.06,<br>0.22	-0.30<br>-0.42,<br>-0.17	0.10<br>-0.03,<br>0.24	0.33<br>0.20,<br>0.45	-0.02<br>-0.16,<br>0.12	0.21<br>0.08,<br>0.34	0.07<br>-0.06,<br>0.20	0.01<br>-0.12,<br>0.14	0.15<br>0.01,<br>0.27	-0.12<br>-0.25,<br>0.01	0.15<br>0.02,<br>0.28	0.15<br>0.02,<br>0.28	-0.03<br>-0.16,<br>0.10
30	-0.30<br>-0.42,<br>-0.17	-0.17<br>-0.30,<br>-0.03	0.01<br>-0.13,<br>0.15	-0.12<br>-0.26,<br>0.02	0.32<br>0.19,<br>0.43	-0.15<br>-0.28,<br>-0.01	-0.36<br>-0.48,<br>-0.24	-0.04<br>-0.17,<br>0.10	-0.34<br>-0.45,<br>-0.22	-0.25<br>0.37,<br>-0.12	-0.04<br>-0.18,<br>0.09	-0.20<br>-0.33,<br>-0.07	0.12<br>-0.01,<br>0.25	-0.23<br>-0.35,<br>-0.10	-0.18<br>-0.31,<br>-0.05	-0.01<br>-0.15,<br>0.12
31	0.21<br>0.07,<br>0.34	0.13<br>-0.01,<br>0.26	0.05<br>-0.09,<br>0.19	0.10<br>-0.04,<br>0.23	-0.25<br>-0.38,<br>-0.12	0.12<br>-0.01,<br>0.26	0.29<br>0.15,<br>0.41	0.13<br>-0.00,<br>0.27	0.39<br>0.27,<br>0.50	0.32<br>0.20,<br>0.44	0.07<br>-0.07,<br>0.20	0.16<br>0.02,<br>0.29	-0.15<br>-0.28,<br>-0.02	0.22<br>0.09,<br>0.34	0.21<br>0.08,<br>0.34	0.01<br>-0.13,<br>0.14
32	0.35<br>0.22,<br>0.46	0.27<br>0.14,<br>0.40	0.01<br>-0.13,<br>0.15	-0.13<br>-0.26,<br>0.01	-0.19<br>-0.32,<br>-0.05	-0.01<br>-0.15,<br>0.12	0.04<br>-0.10,<br>0.18	-0.07<br>-0.21,<br>0.07	0.20<br>0.07,<br>0.33	0.03<br>-0.10,<br>0.17	0.12<br>-0.02,<br>0.25	-0.19<br>-0.32,<br>-0.06)	-0.07<br>-0.20,<br>0.06	-0.19<br>-0.31,<br>-0.05	-0.08<br>-0.21,<br>0.06	-0.10<br>-0.23,<br>0.04

Cardiometabolic traits: A=systolicBP, B=diastolicBP, C=glucose, D=cholesterol, E=HDLcholesterol, F=LDLcholesterol, G=triglycerides, H=heart rate
Anthropometrics traits: 1=weight, 2=tricepsSF, 3=bicepsSF, 4=subscapularSF, 5=suprailiacSF, 6=calfSF, 7=frontthighSF, 8=abdominalSF, 9=calfC, 10=thighC, 11=upperarmC, 12=upperarmflexedC, 13=forearmC, 14=waistC, 15=hipC, 16=biacromialD, 17=bicristalD, 18=humerusD, 19=femurD, 20=height, 21=leglength, 22=bmi, 23=WHR, 24=WHtR, 25=BFmass, 26=BFfreemass, 27=percentageBF, 28=endomorphy, 29=mesomorphy, 30=ectomorphy, 31=factor1, 32=factor2
Abbreviations: BP=blood pressure, HDL=high-density lipoprotein, LDL=low-density lipoprotein, SF=skinfold, C=circumference, D=diameter, BF=body fat

Supplementary table S3. Statistical significance (p-values) of and the size of sex differences in the trait correlations between anthropometric and cardiometabolic traits.

	P-values of sex-differences								Size of sex-differences							
	A	B	C	D	E	F	G	H	A	B	C	D	E	F	G	H
1	0.756	0.475	0.463	0.810	0.040*	0.747	0.036*	0.305	0.02	0.06	-0.07	-0.02	-0.19	0.03	0.20	0.10
2	0.060	0.106	0.543	0.318	0.529	0.328	0.528	0.035*	-0.16	-0.15	-0.06	-0.10	-0.06	-0.09	0.06	0.20
3	0.032*	0.087	0.981	0.690	0.486	0.399	0.113	0.050	-0.19	-0.16	-0.00	0.04	-0.07	0.08	0.15	0.19
4	0.329	0.224	0.872	0.770	0.536	0.557	0.246	0.135	-0.09	-0.11	-0.02	-0.03	-0.06	-0.06	0.11	0.14
5	0.463	0.220	0.954	0.695	0.319	0.549	0.310	0.187	-0.06	-0.11	-0.01	-0.04	-0.10	-0.06	0.10	0.13
6	0.036*	0.045*	0.451	0.976	0.239	0.847	0.184	0.015*	-0.18	-0.18	-0.07	-0.00	-0.11	0.02	0.13	0.23
7	0.022*	0.023*	0.721	0.548	0.153	0.551	0.131	0.043*	-0.20	-0.21	-0.04	-0.06	-0.14	-0.06	0.15	0.19
8	0.455	0.339	0.867	0.772	0.334	0.533	0.224	0.425	-0.07	-0.09	-0.02	-0.03	-0.09	-0.06	0.12	0.08
9	0.527	0.189	0.182	0.890	0.255	0.922	0.152	0.794	0.05	0.12	-0.13	-0.01	-0.11	0.01	0.14	0.03
10	0.798	0.627	0.777	0.801	0.054	0.921	0.199	0.373	-0.02	0.04	-0.03	-0.02	-0.18	0.01	0.12	0.09
11	0.901	0.461	0.523	0.805	0.075	0.545	0.059	0.276	0.01	0.07	-0.06	0.02	-0.16	0.06	0.18	0.11
12	0.637	0.400	0.459	0.849	0.381	0.911	0.123	0.418	0.04	0.08	-0.07	0.02	-0.08	0.01	0.15	0.08
13	0.260	0.139	0.406	0.927	0.038*	0.504	0.142	0.465	0.09	0.14	-0.08	0.01	-0.19	0.07	0.14	0.07
14	0.714	0.632	0.814	0.946	0.033*	0.629	0.017*	0.650	-0.03	-0.04	0.02	0.01	-0.19	0.05	0.22	0.04
15	0.982	0.628	0.861	0.661	0.098	0.418	0.018*	0.113	0.00	0.04	-0.02	0.04	-0.15	0.08	0.23	0.15
16	0.461	0.602	0.270	0.292	0.207	0.086	0.443	0.524	-0.06	0.05	-0.11	0.10	-0.12	0.17	0.08	0.06
17	0.826	0.498	0.486	0.916	0.302	0.507	0.398	0.276	0.02	0.06	-0.07	-0.01	-0.10	0.07	0.08	0.11
18	0.056	0.007**	0.836	0.247	0.003**	0.671	0.667	0.119	0.17	0.26	-0.02	-0.11	-0.29	0.04	0.04	0.15
19	0.887	0.306	0.958	0.724	0.052	0.604	0.033*	0.534	0.01	0.1	-0.01	-0.03	-0.18	0.05	0.21	0.06
20	0.839	0.443	0.515	0.920	0.512	0.477	0.267	0.492	0.02	0.07	-0.06	0.01	-0.06	0.07	0.11	0.07
21	0.730	0.476	0.300	0.442	0.970	0.113	0.184	0.924	0.03	0.07	-0.10	0.07	-0.00	0.15	0.13	0.01
22	0.836	0.822	0.332	0.254	0.039*	0.303	0.049*	0.513	-0.02	-0.02	-0.10	-0.11	-0.19	-0.10	0.18	0.06
23	0.498	0.053	0.448	0.572	0.078	0.777	0.300	0.064	-0.07	-0.19	0.07	-0.06	-0.16	-0.03	0.09	-0.18
24	0.283	0.139	0.538	0.973	0.098	0.868	0.039*	0.978	-0.10	-0.14	0.06	-0.00	-0.15	0.02	0.18	-0.00
25	0.107	0.122	0.558	0.571	0.522	0.483	0.489	0.043*	-0.14	-0.14	-0.06	-0.06	-0.06	-0.07	0.07	0.20
26	0.315	0.443	0.501	0.650	0.222	0.746	0.193	0.076	-0.08	-0.07	-0.07	-0.04	-0.11	-0.03	0.12	0.17
27	0.085	0.060	0.400	0.844	0.020*	0.575	0.029*	0.723	0.14	0.17	-0.08	-0.02	-0.22	0.05	0.21	0.03
28	0.135	0.069	0.783	0.477	0.532	0.303	0.517	0.085	-0.13	-0.17	-0.03	-0.07	-0.06	-0.10	0.06	0.17
29	0.270	0.177	0.905	0.498	0.047*	0.601	0.053	0.890	0.10	0.13	-0.01	-0.07	-0.19	-0.05	0.18	0.01
30	0.636	0.366	0.566	0.408	0.037*	0.392	0.047*	0.812	0.04	0.08	0.06	0.08	0.19	0.08	-0.18	-0.02
31	0.043*	0.039*	0.870	0.548	0.284	0.328	0.434	0.190	-0.18	-0.19	-0.02	-0.06	-0.10	-0.09	0.07	0.13

32	0.106	0.012*	0.272	0.483	0.240	0.078	0.224	0.791	0.15	0.24	-0.11	0.07	-0.11	0.17	0.12	0.03

Cardiometabolic traits: A=systolicBP, B=diastolicBP, C=glucose, D=cholesterol, E=HDLcholesterol, F=LDLcholesterol, G=triglycerides, H=heart rate
Anthropometrics traits: 1=weight, 2=tricepsSF, 3=bicepsSF, 4=subscapularSF, 5=suprailiacSF, 6=calfSF, 7=frontthighSF, 8=abdominalSF, 9=calfC, 10=thighC, 11=upperarmC, 12=upperarmflexedC, 13=forearmC, 14=waistC, 15=hipC, 16=biacromialD, 17=bicristalD, 18=humerusD, 19=femurD, 20=height, 21=leglength, 22=bmi, 23=WHR, 24=WHtR, 25=BFmass, 26=BFfreemass, 27=percentageBF, 28=endomorphy, 29=mesomorphy, 30=ectomorphy, 31=factor1, 32=factor2
Abbreviations: BP=blood pressure, HDL=high-density lipoprotein, LDL=low-density lipoprotein, SF=skinfold, C=circumference, D=diameter, BF=body fat

Supplementary table S4. Additive genetic and unique environmental correlations between anthropometric and metabolic measures with 95% confidence intervals in the pooled data of boys and girls.

	Additive genetic correlations								Unique environmental correlations							
	A	B	C	D	E	F	G	H	A	B	C	D	E	F	G	H
1	0.50 0.36, 0.61	0.40 0.25, 0.53	0.06 -0.09, 0.20	0.02 -0.11, 0.16	-0.24 -0.36, -0.12	0.07 -0.06, 0.20	0.20 0.06, 0.33	-0.01 -0.16, 0.14	0.41 0.22, 0.56	0.06 -0.14, 0.27	0.15 -0.05, 0.34	0.08 -0.14, 0.29	-0.32 -0.49, -0.11	0.19 -0.02, 0.38	0.26 0.06, 0.44	0.10 -0.11, 0.29
2	0.38 0.23, 0.52	0.35 0.19, 0.49	0.08 -0.07, 0.23	0.08 -0.06, 0.22	-0.20 -0.32, -0.07	0.12 -0.01, 0.25	0.26 0.11, 0.39	0.12 -0.03, 0.27	0.31 0.12, 0.49	0.04 -0.16, 0.25	0.09 -0.11, 0.28	0.10 -0.11, 0.31	-0.14 -0.34, 0.07	0.18 -0.03, 0.37	0.11 -0.09, 0.31	-0.06 -0.26, 0.14
3	0.34 0.17, 0.49	0.37 0.21, 0.52	0.04 -0.13, 0.20	0.12 -0.03, 0.26	-0.20 -0.33, -0.06	0.15 0.01, 0.29	0.24 0.09, 0.39	0.15 -0.01, 0.31	0.31 0.12, 0.48	0.06 -0.14, 0.25	0.15 -0.04, 0.34	0.07 -0.14, 0.28	-0.12 -0.31, 0.08	0.12 -0.08, 0.32	0.12 -0.07, 0.31	0.04 -0.15, 0.24
4	0.40 0.25, 0.53	0.35 0.20, 0.49	0.06 -0.10, 0.21	0.08 -0.06, 0.21	-0.23 -0.35, -0.10	0.14 0.01, 0.27	0.22 0.08, 0.36	0.10 -0.05, 0.25	0.34 0.14, 0.51	0.11 -0.10, 0.31	0.10 -0.10, 0.29	0.12 -0.10, 0.32	-0.20 -0.40, 0.01	0.14 -0.07, 0.34	0.30 0.10, 0.47	0.04 -0.16, 0.24
5	0.37 0.21, 0.51	0.34 0.19, 0.49	0.07 -0.09, 0.22	0.07 -0.07, 0.20	-0.17 -0.29, -0.04	0.12 -0.01, 0.25	0.21 0.06, 0.35	0.07 -0.08, 0.22	0.31 0.11, 0.48	-0.04 -0.24, 0.17	0.04 -0.16, 0.24	0.09 -0.13, 0.30	-0.27 -0.46, -0.07	0.12 -0.10, 0.32	0.21 0.01, 0.40	-0.01 -0.21, 0.19
6	0.33 0.16, 0.47	0.31 0.14, 0.46	0.06 -0.10, 0.22	0.04 -0.10, 0.18	-0.24 -0.37, -0.11	0.10 -0.04, 0.24	0.21 0.06, 0.35	0.12 -0.03, 0.27	0.40 0.22, 0.55	0.17 -0.03, 0.36	0.00 -0.19, 0.19	0.16 -0.05, 0.36	0.02 -0.18, 0.22	0.16 -0.05, 0.35	0.16 -0.03, 0.35	0.05 -0.15, 0.24
7	0.39 0.24, 0.53	0.27 0.11, 0.42	0.01 -0.15, 0.16	0.07 -0.07, 0.20	-0.20 -0.32, -0.07	0.12 -0.01, 0.25	0.20 0.05, 0.33	0.15 -0.00, 0.29	0.20 0.00, 0.39	0.24 0.04, 0.43	0.12 -0.08, 0.31	0.18 -0.03, 0.38	-0.01 -0.22, 0.19	0.09 -0.12, 0.29	0.11 -0.09, 0.30	0.10 -0.10, 0.29
8	0.37 0.20, 0.51	0.32 0.16, 0.47	0.10 -0.06, 0.25	0.07 -0.07, 0.21	-0.21 -0.34, -0.08	0.13 -0.00, 0.26	0.23 0.07, 0.37	0.11 -0.04, 0.26	0.33 0.13, 0.50	0.11 -0.10, 0.31	0.05 -0.15, 0.25	0.09 -0.12, 0.30	-0.06 -0.27, 0.15	0.10 -0.11, 0.31	0.13 -0.07, 0.33	-0.07 -0.26, 0.14
9	0.48 0.33, 0.61	0.31 0.15, 0.46	0.00 -0.16, 0.16	-0.02 -0.17, 0.12	-0.29 -0.41, -0.16	0.08 -0.06, 0.22	0.18 0.02, 0.32	0.02 -0.13, 0.18	0.16 -0.04, 0.35	0.11 -0.09, 0.31	0.16 -0.04, 0.34	0.07 -0.14, 0.28	-0.18 -0.38, 0.02	-0.01 -0.22, 0.19	0.22 0.02, 0.40	-0.04 -0.24, 0.16
10	0.39 0.22, 0.54	0.36 0.19, 0.51	0.08 -0.09, 0.24	-0.00 -0.15, 0.14	-0.30 -0.42, -0.17	0.04 -0.10, 0.19	0.15 -0.01, 0.30	0.00 -0.16, 0.16	0.41 0.24, 0.56	0.09 -0.11, 0.28	0.12 -0.07, 0.31	0.06 -0.15, 0.27	-0.29 -0.47, -0.10	0.17 -0.03, 0.36	0.28 0.09, 0.45	0.03 -0.16, 0.23

11	0.46 0.30, 0.59	0.31 0.14, 0.46	0.07 -0.10, 0.24	0.07 -0.08, 0.22	-0.28 -0.40, -0.14	0.11 -0.04, 0.25	0.26 0.10, 0.40	-0.01 -0.18, 0.15	0.33 0.15, 0.50	0.17 -0.03, 0.36	0.10 -0.09, 0.29	-0.00 -0.21, 0.21	-0.23 -0.41, -0.03	0.11 -0.09, 0.31	0.14 -0.05, 0.33	0.02 -0.17, 0.22
12	0.47 0.30, 0.61	0.26 0.08, 0.43	0.03 -0.15, 0.21	0.05 -0.11, 0.21	-0.26 -0.40, -0.11	0.09 -0.06, 0.24	0.22 0.04, 0.37	-0.05 -0.23, 0.12	0.26 0.08, 0.43	0.19 -0.01, 0.37	0.08 -0.11, 0.26	-0.01 -0.21, 0.20	-0.23 0.42, -0.04	0.09 -0.11, 0.29	0.22 0.03, 0.39	0.02 -0.18, 0.21
13	0.43 0.27, 0.55	0.33 0.18, 0.47	0.07 -0.09, 0.22	0.05 -0.09, 0.18	-0.29 -0.41, -0.17	0.10 -0.04, 0.23	0.19 0.04, 0.33	-0.04 -0.19, 0.12	0.44 0.26, 0.59	0.08 -0.13, 0.29	0.05 -0.15, 0.25	0.04 -0.17, 0.26	-0.19 -0.39, 0.02	0.17 -0.04, 0.36	0.20 -0.00, 0.39	0.01 -0.20, 0.21
14	0.43 0.27, 0.56	0.39 0.23, 0.53	0.11 -0.05, 0.26	0.07 -0.07, 0.21	-0.36 -0.47, -0.23	0.14 -0.00, 0.27	0.27 0.12, 0.41	0.10 -0.05, 0.25	0.37 0.19, 0.54	0.07 -0.13, 0.28	-0.03 -0.22, 0.17	0.07 -0.15, 0.28	-0.27 -0.46, -0.07	0.16 -0.05, 0.36	0.38 0.20, 0.54	0.01 -0.19, 0.21
15	0.49 0.36, 0.60	0.40 0.26, 0.53	0.10 -0.05, 0.24	0.03 -0.10, 0.16	-0.27 -0.38, -0.15	0.09 -0.04, 0.21	0.20 0.06, 0.33	0.01 -0.14, 0.15	0.38 0.19, 0.54	0.13 -0.08, 0.33	0.17 -0.03, 0.36	0.14 -0.07, 0.35	-0.15 -0.34, 0.06	0.17 -0.04, 0.36	0.19 -0.01, 0.37	0.06 -0.14, 0.26
16	0.42 0.26, 0.56	0.29 0.13, 0.44	0.05 -0.10, 0.21	-0.14 -0.29, -0.00	-0.14 -0.27, -0.01	-0.13 -0.27, 0.00	0.08 -0.08, 0.23	-0.08 -0.23, 0.08	0.30 0.10, 0.47	0.16 -0.05, 0.36	0.03 -0.17, 0.23	0.09 -0.13, 0.31	-0.17 -0.36, 0.04	0.15 -0.06, 0.35	0.05 -0.15, 0.25	0.09 -0.11, 0.29
17	0.28 0.11, 0.43	0.26 0.09, 0.42	0.09 -0.07, 0.25	-0.08 -0.22, 0.06	-0.17 -0.30, -0.03	-0.01 -0.15, 0.13	0.05 -0.11, 0.20	-0.00 -0.16, 0.15	0.29 0.10, 0.47	-0.02 -0.22, 0.19	0.07 -0.13, 0.26	0.01 -0.20, 0.22	-0.23 -0.42, -0.03	0.12 -0.09, 0.31	0.19 -0.01, 0.38	0.02 -0.17, 0.22
18	0.31 0.14, 0.46	0.19 0.02, 0.35	0.03 -0.13, 0.19	0.04 -0.10, 0.17	-0.18 -0.31, -0.05	0.08 -0.05, 0.22	0.12 -0.03, 0.27	-0.08 -0.23, 0.08	0.27 0.07, 0.44	0.16 -0.05, 0.35	0.12 -0.07, 0.31	-0.15 -0.35, 0.07	-0.08 -0.29, 0.12	-0.10 -0.30, 0.11	0.07 -0.13, 0.27	-0.06 -0.26, 0.14
19	0.40 0.22, 0.57	0.31 0.12, 0.48	0.09 -0.11, 0.27	0.06 -0.11, 0.22	-0.22 -0.36, -0.06	0.10 -0.06, 0.26	0.16 -0.03, 0.33	-0.05 -0.24, 0.13	0.11 -0.08, 0.30	0.04 -0.15, 0.23	0.05 -0.14, 0.24	0.02 -0.18, 0.23	-0.19 -0.37, 0.01	0.01 -0.19, 0.21	0.22 0.03, 0.40	0.05 -0.14, 0.24
20	0.29 0.15, 0.43	0.24 0.10, 0.38	0.06 -0.09, 0.19	-0.12 -0.24, 0.00	-0.06 -0.18, 0.06	-0.09 -0.22, 0.03	-0.02 -0.16, 0.12	-0.08 -0.22, 0.06	0.30 0.10, 0.48	0.03 -0.18, 0.24	0.14 -0.06, 0.33	-0.04 -0.24, 0.18	-0.35 -0.52, -0.16	0.12 -0.08, 0.32	0.06 -0.14, 0.26	0.19 -0.01, 0.38
21	0.24 0.08, 0.39	0.20 0.05, 0.35	0.06 -0.09, 0.20	-0.07 -0.20, 0.06	0.05 -0.08, 0.18	-0.07 -0.20, 0.06	-0.11 -0.25, 0.04	-0.02 -0.16, 0.13	0.23 0.02, 0.41	0.01 -0.20, 0.22	0.07 -0.13, 0.26	-0.06 -0.27, 0.16	-0.38 -0.54, -0.19	0.05 -0.15, 0.25	0.03 -0.17, 0.23	0.07 -0.13, 0.27
22	0.47 0.34, 0.59	0.33 0.19, 0.46	0.02 -0.13, 0.16	0.12 -0.01, 0.24	-0.25 -0.36, -0.13	0.17 0.04, 0.29	0.28 0.14, 0.41	0.03 -0.11, 0.17	0.33 0.13, 0.50	0.06 -0.14, 0.27	0.09 -0.11, 0.28	0.10 -0.11, 0.31	-0.20 -0.39, 0.00	0.14 -0.07, 0.33	0.26 0.06, 0.44	0.04 -0.17, 0.24

23	-0.03 -0.27, 0.18	0.06 -0.16, 0.27	0.03 -0.16, 0.23	0.14 -0.03, 0.32	-0.29 -0.43, -0.13	0.18 0.01, 0.34	0.26 0.07, 0.43	0.27 0.08, 0.46	0.14 -0.05, 0.32	-0.02 -0.21, 0.17	-0.19 -0.37, -0.00	-0.07 -0.27, 0.13	-0.26 -0.44, -0.07	0.04 -0.16, 0.24	0.33 0.14, 0.49	-0.06 -0.24, 0.13
24	0.30 0.13, 0.45	0.30 0.13, 0.45	0.08 -0.08, 0.24	0.15 0.01, 0.29	-0.35 -0.46, -0.22	0.22 0.08, 0.35	0.32 0.18, 0.45	0.15 -0.00, 0.30	0.33 0.14, 0.50	0.07 -0.13, 0.27	-0.08 -0.27, 0.12	0.07 -0.15, 0.27	-0.21 -0.40, -0.01	0.13 -0.07, 0.33	0.39 0.21, 0.55	-0.03 -0.22, 0.17
25	0.40 0.25, 0.53	0.35 0.20, 0.49	0.06 -0.09, 0.21	0.08 -0.06, 0.21	-0.22 -0.34, -0.10	0.13 -0.00, 0.25	0.25 0.11, 0.38	0.10 -0.05, 0.24	0.32 0.13, 0.49	0.08 -0.13, 0.28	0.09 -0.11, 0.28	0.10 -0.12, 0.30	-0.18 -0.37, 0.02	0.16 -0.05, 0.35	0.19 -0.01, 0.38	-0.02 -0.22, 0.18
26	0.46 0.32, 0.58	0.38 0.24, 0.52	0.06 -0.09, 0.21	0.06 -0.07, 0.19	-0.24 -0.36, -0.12	0.11 -0.02, 0.24	0.25 0.11, 0.38	0.06 -0.08, 0.21	0.38 0.19, 0.54	0.08 -0.13, 0.29	0.12 -0.08, 0.31	0.10 -0.12, 0.31	-0.25 -0.43, -0.04	0.18 -0.02, 0.38	0.23 0.03, 0.41	0.02 -0.18, 0.22
27	0.46 0.32, 0.59	0.32 0.17, 0.47	0.03 -0.13, 0.18	-0.03 -0.16, 0.11	-0.18 -0.30, -0.05	-0.00 -0.14, 0.13	0.11 -0.04, 0.26	-0.11 -0.26, 0.05	0.29 0.09, 0.47	0.06 -0.15, 0.27	0.15 -0.06, 0.33	0.03 -0.19, 0.25	-0.25 -0.44, -0.04	0.12 -0.09, 0.32	0.15 -0.06, 0.34	0.15 -0.06, 0.34
28	0.37 0.22, 0.50	0.32 0.17, 0.47	0.07 -0.09, 0.21	0.08 -0.05, 0.22	-0.20 -0.32, -0.07	0.14 0.01, 0.26	0.24 0.10, 0.37	0.10 -0.05, 0.24	0.32 0.13, 0.50	0.04 -0.17, 0.25	0.06 -0.14, 0.26	0.11 -0.11, 0.32	-0.19 -0.38, 0.02	0.14 -0.07, 0.33	0.20 -0.00, 0.39	-0.03 -0.23, 0.17
29	0.38 0.22, 0.52	0.18 0.01, 0.33	0.00 -0.16, 0.16	0.15 0.01, 0.29	-0.29 -0.40, -0.16	0.20 0.07, 0.33	0.24 0.09, 0.38	0.04 -0.12, 0.19	0.10 -0.10, 0.29	0.14 -0.06, 0.34	0.05 -0.14, 0.25	-0.03 -0.24, 0.18	-0.11 -0.31, 0.09	-0.08 -0.27, 0.13	0.26 0.06, 0.43	-0.13 -0.32, 0.07
30	-0.41 -0.53, -0.27	-0.29 -0.43, -0.15	-0.01 -0.16, 0.13	-0.15 -0.27, -0.02	0.28 0.16, 0.39	-0.20 -0.32, -0.08	-0.30 -0.42, -0.16	-0.09 -0.23, 0.05	-0.23 -0.41, -0.03	-0.08 -0.29, 0.13	-0.07 -0.26, 0.13	-0.19 -0.39, 0.03	0.07 -0.14, 0.27	-0.15 -0.34, 0.06	-0.27 -0.44, -0.07	0.11 -0.10, 0.30
31	0.35 0.19, 0.48	0.31 0.15, 0.45	0.07 -0.09, 0.22	0.10 -0.03, 0.24	-0.26 -0.38, -0.13	0.16 0.03, 0.29	0.28 0.14, 0.41	0.12 -0.03, 0.26	0.35 0.16, 0.52	0.11 -0.10, 0.31	0.08 -0.12, 0.28	0.15 -0.07, 0.35	-0.10 -0.31, 0.11	0.16 -0.05, 0.36	0.23 0.03, 0.41	-0.01 -0.21, 0.19
32	0.33 0.17, 0.47	0.22 0.07, 0.37	0.07 -0.09, 0.22	-0.10 -0.23, 0.04	-0.10 -0.23, 0.03	-0.07 -0.20, 0.06	-0.02 -0.17, 0.12	-0.12 -0.26, 0.03	0.23 0.02, 0.41	0.04 -0.17, 0.25	0.09 -0.12, 0.28	-0.10 -0.31, 0.12	-0.34 -0.51, -0.14	0.02 -0.19, 0.23	0.12 -0.09, 0.31	0.06 -0.14, 0.26

Cardiometabolic traits: A=systolicBP, B=diastolicBP, C=glucose, D=cholesterol, E=HDLcholesterol, F=LDLcholesterol, G=triglycerides, H=heart rate
Anthropometrics traits: 1=weight, 2=tricepsSF, 3=bicepsSF, 4=subscapularSF, 5=suprailiacSF, 6=calfSF, 7=frontthighSF, 8=abdominalSF, 9=calfC, 10=thighC, 11=upperarmC, 12=upperarmflexedC, 13=forearmC, 14=waistC, 15=hipC, 16=biacromialD, 17=bicristalD, 18=humerusD, 19=femurD, 20=height, 21=leglength, 22=bmi, 23=WHR, 24=WHtR, 25=BFmass, 26=BFfreemass, 27=percentageBF, 28=endomorphy, 29=mesomorphy, 30=ectomorphy, 31=factor1, 32=factor2
Abbreviations: BP=blood pressure, HDL=high-density lipoprotein, LDL=low-density lipoprotein, SF=skinfold, C=circumference, D=diameter, BF=body fat

Supplementary table S5. Additive genetic correlations between anthropometric and metabolic measures with 95% confidence intervals in boys and girls.

	Boys								Girls							
	A	B	C	D	E	F	G	H	A	B	C	D	E	F	G	H
1	0.54 0.38, 0.68	0.44 0.25, 0.61	0.11 -0.11, 0.32	0.08 -0.09, 0.25	-0.28 -0.42, -0.12	0.16 -0.02, 0.33	0.31 0.13, 0.48	0.08 -0.11, 0.27	0.42 0.17, 0.62	0.44 0.20, 0.67	0.03 -0.19, 0.24	-0.04 -0.24, 0.15	-0.20 -0.37, -0.01	-0.01 -0.23, 0.19	0.07 -0.16, 0.27	-0.06 -0.29, 0.19
2	0.33 0.13, 0.51	0.30 0.09, 0.50	0.12 -0.09, 0.33	0.07 -0.11, 0.26	-0.19 -0.35, -0.01	0.10 -0.08, 0.28	0.25 0.05, 0.44	0.27 0.08, 0.45	0.45 0.21, 0.66	0.40 0.17, 0.61	0.02 -0.19, 0.22	0.09 -0.10, 0.27	-0.22 -0.39, -0.04	0.14 -0.04, 0.31	0.26 0.06, 0.44	-0.05 -0.26, 0.17
3	0.27 0.06, 0.46	0.29 0.08, 0.49	0.10 -0.12, 0.32	0.16 -0.03, 0.34	-0.18 -0.35, -0.01	0.20 0.01, 0.37	0.27 0.07, 0.47	0.27 0.08, 0.45	0.46 0.18, 0.70	0.47 0.22, 0.71	-0.05 -0.27, 0.16	0.07 -0.14, 0.28	-0.21 -0.39, -0.02	0.12 -0.08, 0.32	0.22 -0.00, 0.42	0.02 -0.22, 0.26
4	0.37 0.17, 0.55	0.37 0.16, 0.57	0.10 -0.13, 0.31	0.13 -0.06, 0.31	-0.20 -0.36, -0.02	0.16 -0.03, 0.33	0.27 0.06, 0.46	0.19 -0.01, 0.37	0.43 0.21, 0.62	0.33 0.10, 0.52	0.02 -0.19, 0.21	0.02 -0.16, 0.19	-0.25 -0.41, -0.08	0.12 -0.05, 0.29	0.18 -0.02, 0.35	0.02 -0.19, 0.23
5	0.38 0.18, 0.55	0.33 0.11, 0.54	0.13 -0.09, 0.34	0.10 -0.09, 0.28	-0.18 -0.34, 0.00	0.14 -0.05, 0.33	0.26 0.05, 0.45	0.14 -0.06, 0.33	0.35 0.08, 0.57	0.36 0.13, 0.57	-0.00 -0.22, 0.20	0.03 -0.16, 0.21	-0.16 -0.34, 0.02	0.15 -0.03, 0.33	0.15 -0.06, 0.34	-0.00 -0.22, 0.22
6	0.28 0.07, 0.47	0.22 -0.00, 0.44	0.08 -0.14, 0.30	0.08 -0.12, 0.26	-0.24 -0.40, -0.06	0.12 -0.07, 0.30	0.24 0.02, 0.43	0.25 0.05, 0.43	0.39 0.12, 0.59	0.39 0.16, 0.59	0.03 -0.18, 0.23	0.01 -0.19, 0.20	-0.25 -0.42, -0.06	0.09 -0.10, 0.27	0.18 -0.02, 0.38	-0.02 -0.25, 0.20
7	0.29 0.09, 0.47	0.18 -0.04, 0.37	0.04 -0.17, 0.25	0.10 -0.08, 0.28	-0.18 -0.34, -0.01	0.13 -0.05, 0.30	0.23 0.03, 0.42	0.26 0.07, 0.43	0.53 0.30, 0.72	0.38 0.15, 0.57	-0.04 -0.25, 0.16	0.03 -0.17, 0.21	-0.21 -0.39, -0.03	0.11 -0.07, 0.29	0.16 -0.04, 0.35	0.01 -0.21, 0.23
8	0.41 0.22, 0.58	0.33 0.12, 0.53	0.19 -0.03, 0.39	0.14 -0.05, 0.32	-0.19 -0.35, -0.01	0.19 -0.00, 0.37	0.25 0.04, 0.44	0.15 -0.05, 0.33	0.31 0.01, 0.54	0.30 0.04, 0.52	-0.02 -0.25, 0.19	-0.01 -0.21, 0.18	-0.24 -0.41, -0.06	0.15 -0.04, 0.34	0.21 -0.00, 0.40	0.09 -0.14, 0.32
9	0.58 0.40, 0.73	0.37 0.16, 0.57	-0.00 -0.22, 0.22	0.03 -0.17, 0.22	-0.30 -0.46, -0.13	0.17 -0.03, 0.36	0.26 0.05, 0.45	0.08 -0.13, 0.28	0.34 0.06, 0.55	0.26 0.01, 0.47	-0.01 -0.24, 0.19	-0.09 -0.28, 0.11	-0.27 -0.44, -0.09	-0.02 -0.23, 0.18	0.08 -0.14, 0.28	-0.05 -0.29, 0.18
10	0.43 0.25, 0.59	0.38 0.19, 0.56	0.15 -0.06, 0.35	0.04 -0.14, 0.22	-0.32 -0.46, -0.15	0.09 -0.09, 0.27	0.24 0.04, 0.42	0.06 -0.13, 0.25	0.31 -0.07, 0.56	0.28 -0.05, 0.53	-0.04 -0.31, 0.19	-0.05 -0.29, 0.18	-0.25 -0.44, -0.03	-0.01 -0.26, 0.23	0.04 -0.22, 0.27	-0.11 -0.39, 0.16
11	0.55 0.37, 0.70	0.43 0.22, 0.62	0.11 -0.12, 0.34	0.16 -0.04, 0.35	-0.30 -0.47, -0.12	0.23 0.02, 0.42	0.36 0.15, 0.55	0.10 -0.12, 0.30	0.34 0.04, 0.56	0.16 -0.15, 0.40	0.02 -0.22, 0.23	-0.03 -0.25, 0.18	-0.25 -0.42, -0.05	-0.02 -0.29, 0.21	0.15 -0.08, 0.35	-0.13 0.37, 0.10

12	0.56 0.37, 0.72	0.39 0.17, 0.59	0.06 -0.17, 0.29	0.16 -0.04, 0.35	-0.25 -0.42, -0.07	0.20 0.00, 0.40	0.31 0.09, 0.50	0.01 -0.21, 0.22	0.34 -0.02, 0.59	0.08 -0.32, 0.36	-0.01 -0.27, 0.23	-0.08 -0.33, 0.16	-0.27 -0.46, -0.05	-0.01 -0.59, 0.85	0.11 -0.15, 0.34	-0.16 -0.43, 0.11
13	0.53 0.36, 0.67	0.46 0.27, 0.64	0.13 -0.07, 0.34	0.14 -0.04, 0.32	-0.31 -0.46, -0.15	0.19 0.02, 0.36	0.27 0.07, 0.45	0.02 -0.17, 0.21	0.26 -0.06, 0.49	0.15 -0.15, 0.38	-0.05 -0.30, 0.17	-0.08 -0.30, 0.12	-0.26 -0.43, -0.07	-0.02 -0.23, 0.18	0.10 -0.13, 0.31	-0.11 -0.35, 0.12
14	0.47 0.29, 0.63	0.41 0.20, 0.60	0.17 -0.06, 0.38	0.14 -0.05, 0.32	-0.39 -0.53, -0.22	0.22 0.03, 0.40	0.40 0.20, 0.56	0.15 -0.04, 0.34	0.36 0.06, 0.57	0.36 0.10, 0.57	0.10 -0.13, 0.33	-0.01 -0.22, 0.19	-0.33 -0.49, -0.14	0.08 -0.13, 0.28	0.11 -0.22, 0.35	0.04 -0.19, 0.27
15	0.53 0.37, 0.66	0.44 0.26, 0.61	0.16 -0.05, 0.35	0.11 -0.07, 0.28	-0.29 -0.44, -0.13	0.18 0.01, 0.35	0.31 0.13, 0.49	0.12 -0.07, 0.30	0.43 0.18, 0.62	0.36 0.13, 0.55	0.01 -0.21, 0.20	-0.05 -0.25, 0.13	-0.25 -0.41, -0.07	-0.02 -0.21, 0.17	0.07 -0.14, 0.27	-0.13 -0.35, 0.09
16	0.45 0.25, 0.63	0.31 0.10, 0.51	0.06 -0.17, 0.28	0.04 -0.14, 0.22	-0.14 -0.31, 0.03	0.05 -0.13, 0.23	0.17 -0.05, 0.37	-0.04 -0.23, 0.16	0.39 0.12, 0.61	0.27 0.01, 0.50	0.10 -0.12, 0.33	-0.35 -0.59, -0.14	-0.13 -0.32, 0.07	-0.32 -0.53, -0.12	-0.03 -0.29, 0.20	-0.14 -0.39, 0.09
17	0.36 0.16, 0.54	0.27 0.06, 0.48	0.05 -0.17, 0.26	-0.00 -0.19, 0.18	-0.17 -0.33, 0.00	0.06 -0.12, 0.24	0.15 -0.06, 0.34	0.07 -0.12, 0.26	0.14 -0.24, 0.41	0.22 -0.08, 0.47	0.15 -0.08, 0.37	-0.15 -0.37, 0.07	-0.15 -0.34, 0.07	-0.09 -0.30, 0.12	-0.06 -0.31, 0.16	-0.09 -0.34, 0.16
18	0.36 0.16, 0.53	0.34 0.13, 0.52	0.09 -0.13, 0.29	0.07 0.11, 0.25	-0.27 -0.43, -0.10	0.18 -0.00, 0.36	0.16 -0.05, 0.35	-0.01 -0.21, 0.18	0.21 -0.11, 0.48	-0.04 -0.38, 0.23	-0.07 -0.32, 0.16	-0.02 -0.25, 0.19	-0.05 -0.25, 0.16	-0.03 -0.30, 0.23	0.08 -0.16, 0.30	-0.16 -0.38, 0.09
19	0.44 0.25, 0.62	0.35 0.15, 0.54	0.18 -0.03, 0.38	0.12 -0.07, 0.30	-0.26 -0.41, -0.09	0.17 -0.02, 0.34	0.31 0.11, 0.49	0.05 -0.14, 0.24	0.21 -0.94, 0.64	0.99 -1.00, NA	-0.12 -0.61, 0.24	0.02 -0.87, 0.51	-0.07 -0.39, 0.29	0.04 -0.36, 0.45	-0.15 -0.56, 0.20	-0.87 -1.00, -0.14
20	0.27 0.03, 0.48	0.26 0.06, 0.46	0.01 -0.17, 0.19	-0.14 -0.31, 0.03	-0.03 -0.20, 0.14	-0.15 -0.32, 0.02	-0.12 -0.36, -0.12	-0.15 -0.35, 0.04	0.30 0.12, 0.47	0.22 0.02, 0.40	0.09 -0.11, 0.28	-0.10 -0.26, 0.07	-0.08 -0.24, 0.08	-0.03 -0.20, 0.14	0.10 -0.11, 0.32	-0.00 -0.19, 0.17
21	0.25 0.05, 0.44	0.18 -0.03, 0.39	0.06 -0.15, 0.27	-0.02 -0.20, 0.15	0.03 -0.15, 0.21	0.04 -0.13, 0.22	0.01 -0.20, 0.21	0.03 -0.16, 0.22	0.22 -0.03, 0.43	0.21 -0.00, 0.42	0.04 -0.15, 0.22	-0.11 -0.29, 0.07	0.08 -0.11, 0.27	-0.16 -0.33, 0.02	-0.22 -0.41, -0.03	-0.05 -0.26, 0.15
22	0.44 0.22, 0.63	0.31 0.08, 0.51	0.00 -0.20, 0.19	0.10 -0.11, 0.28	-0.23 -0.39, -0.06	0.17 -0.01, 0.34	0.19 -0.01, 0.37	-0.04 -0.25, 0.17	0.50 0.33, 0.64	0.35 0.16, 0.52	0.02 -0.18, 0.22	0.14 -0.05, 0.32	-0.27 -0.41, -0.10	0.15 -0.02, 0.32	0.36 NA, 0.53	0.09 -0.10, 0.27
23	-0.04 -0.36, 0.25	0.01 -0.33, 0.34	0.04 -0.24, 0.33	0.17 -0.08, 0.45	-0.34 -0.53, -0.12	0.19 -0.05, 0.44	0.23 -0.06, 0.46	0.12 -0.14, 0.38	-0.02 -0.38, 0.30	0.10 -0.22, 0.41	0.04 -0.18, 0.28	0.11 -0.12, 0.34	-0.25 -0.44, -0.04	0.17 -0.05, 0.38	0.28 0.04, 0.50	0.41 0.15, 0.71

24	0.32 0.12, 0.50	0.28 0.07, 0.49	0.12 -0.10, 0.33	0.21 0.01, 0.39	-0.35 -0.49, -0.18	0.25 0.06, 0.42	0.37 0.17, 0.54	0.16 -0.04, 0.35	0.29 -0.00, 0.51	0.30 0.05, 0.52	0.05 -0.16, 0.25	0.10 -0.10, 0.29	-0.35 -0.50, -0.17	0.19 -0.00, 0.37	0.28 0.07, 0.46	0.13 -0.10, 0.35
25	0.35 0.15, 0.53	0.33 0.13, 0.53	0.09 -0.12, 0.30	0.10 -0.08, 0.29	-0.20 -0.36, -0.03	0.13 -0.05, 0.31	0.26 0.05, 0.45	0.23 0.03, 0.41	0.48 0.26, 0.66	0.37 0.16, 0.56	0.02 -0.18, 0.21	0.04 -0.14, 0.21	-0.25 -0.40, -0.08	0.12 -0.06, 0.28	0.24 0.05, 0.41	-0.04 -0.24, 0.16
26	0.44 0.26, 0.60	0.38 0.19, 0.57	0.09 -0.12, 0.30	0.10 -0.08, 0.27	-0.24 -0.40, -0.08	0.14 -0.04, 0.31	0.30 0.10, 0.47	0.18 -0.01, 0.36	0.48 0.27, 0.66	0.39 0.17, 0.58	0.01 -0.19, 0.20	0.01 -0.17, 0.19	-0.25 -0.40, -0.07	0.08 -0.10, 0.25	0.19 -0.00, 0.37	-0.07 -0.27, 0.14
27	0.57 0.40, 0.71	0.37 0.18, 0.55	0.06 -0.16, 0.27	0.03 -0.15, 0.21	-0.24 -0.39, -0.07	0.07 -0.11, 0.25	0.24 0.05, 0.43	-0.04 -0.23, 0.16	0.30 -0.01, 0.53	0.25 -0.01, 0.48	0.02 -0.20, 0.24	-0.09 -0.31, 0.11	-0.09 -0.28, 0.12	-0.15 -0.96, 0.06	-0.06 -0.30, 0.16	-0.10 -0.35, 0.17
28	0.33 0.08, 0.52	0.25 0.05, 0.45	0.13 -0.11, 0.36	0.13 -0.06, 0.31	-0.20 -0.38, -0.01	0.14 -0.05, 0.32	0.25 0.05, 0.44	0.20 -0.01, 0.40	0.44 0.19, 0.65	0.37 0.18, 0.55	0.03 -0.89, 0.23	0.09 -0.08, 0.26	-0.22 -0.40, 0.42	0.18 0.00, 0.34	0.23 0.04, 0.40	0.00 -0.22, 0.23
29	0.49 0.31, 0.64	0.35 0.13, 0.55	0.04 -0.17, 0.25	0.18 -0.01, 0.36	-0.31 -0.46, -0.15	0.24 0.05, 0.43	0.34 0.15, 0.52	0.05 -0.15, 0.24	0.25 -0.03, 0.49	0.14 -0.15, 1.00	-0.05 -0.27, 0.16	0.12 -0.08, 0.32	-0.25 -0.42, -0.07	0.18 -0.02, 0.37	0.14 -0.07, 0.34	0.01 -0.22, 0.23
30	-0.42 -0.57, -0.24	-0.30 -0.48, -0.11	-0.02 -0.22, 0.19	-0.16 -0.33, 0.01	0.30 0.14, 0.44	-0.19 -0.35, -0.01	-0.39 -0.55, -0.20	-0.10 -0.28, 0.08	-0.40 -0.60, -0.17	-0.28 -0.48, -0.06	-0.00 -0.19, 0.19	-0.14 -0.31, 0.04	0.26 0.09, 0.42	-0.22 -0.38, -0.04	-0.21 -0.38, -0.02	-0.07 -0.27, 0.14
31	0.31 0.11, 0.49	0.27 0.06, 0.47	0.14 -0.09, 0.37	0.13 -0.05, 0.31	-0.24 -0.40, -0.07	0.15 -0.03, 0.33	0.28 0.08, 0.46	0.20 0.01, 0.37	0.40 0.15, 0.61	0.35 0.12, 0.56	0.02 -0.21, 0.24	0.07 -0.11, 0.25	-0.28 -0.44, -0.11	0.20 0.02, 0.37	0.28 0.09, 0.46	0.03 -0.18, 0.24
32	0.43 0.24, 0.60	0.31 0.11, 0.51	0.07 -0.14, 0.28	-0.02 -0.19, 0.16	-0.13 -0.30, 0.04	0.07 -0.11, 0.25	0.09 -0.11, 0.29	-0.08 -0.26, 0.11	0.16 -0.16, 0.41	0.10 -0.18, 0.34	0.10 -0.13, 0.31	-0.29 -0.49, -0.09	-0.04 -0.23, 0.17	-0.30 -0.96, -0.10	-0.21 -0.45, 0.02	-0.18 -0.42, 0.05

Cardiometabolic traits: A=systolicBP, B=diastolicBP, C=glucose, D=cholesterol, E=HDLcholesterol, F=LDLcholesterol, G=triglycerides, H=heart rate
Anthropometrics traits: 1=weight, 2=tricepsSF, 3=bicepsSF, 4=subscapularSF, 5=suprailiacSF, 6=calfSF, 7=frontthighSF, 8=abdominalSF, 9=calfC, 10=thighC, 11=upperarmC, 12=upperarmflexedC, 13=forearmC, 14=waistC, 15=hipC, 16=biacromialD, 17=bicristalD, 18=humerusD, 19=femurD, 20=height, 21=leglength, 22=bmi, 23=WHR, 24=WHtR, 25=BFmass, 26=BFfreemass, 27=percentageBF, 28=endomorphy, 29=mesomorphy, 30=ectomorphy, 31=factor1, 32=factor2
Abbreviations: BP=blood pressure, HDL=high-density lipoprotein, LDL=low-density lipoprotein, SF=skinfold, C=circumference, D=diameter, BF=body fat

Supplementary table S6. Unique environmental correlations between anthropometric and metabolic measures with 95% confidence intervals in boys and girls.

	Boys								Girls							
	A	B	C	D	E	F	G	H	A	B	C	D	E	F	G	H
1	0.18 -0.11, 0.45	0.00 -0.30, 0.31	-0.09 -0.35, 0.19	-0.21 -0.47, 0.09	-0.59 -0.75, -0.36	-0.05 -0.33, 0.23	0.18 -0.11, 0.44	0.11 -0.18, 0.39	0.52 0.28, 0.70	-0.02 -0.32, 0.29	0.36 0.06, 0.59	0.28 -0.04, 0.55	-0.20 -0.47, 0.10	0.38 0.07, 0.63	0.34 0.05, 0.57	0.00 -0.30, 0.30
2	0.19 -0.11, 0.46	-0.05 -0.34, 0.25	-0.12 -0.37, 0.16	-0.03 -0.32, 0.27	-0.22 -0.48, 0.08	0.07 -0.22, 0.34	0.17 -0.12, 0.43	-0.05 -0.33, 0.24	0.40 0.14, 0.61	0.12 -0.17, 0.39	0.39 0.13, 0.61	0.23 -0.07, 0.49	-0.08 -0.36, 0.20	0.28 -0.01, 0.53	0.06 -0.22, 0.33	-0.07 -0.33, 0.21
3	0.25 -0.05, 0.51	0.01 -0.28, 0.31	0.02 -0.25, 0.28	0.03 -0.27, 0.32	-0.14 -0.42, 0.15	0.15 -0.14, 0.41	0.19 -0.09, 0.45	0.09 -0.20, 0.37	0.34 0.08, 0.55	0.08 -0.20, 0.35	0.35 0.09, 0.56	0.11 -0.19, 0.39	-0.12 -0.38, 0.16	0.11 -0.18, 0.38	0.06 -0.21, 0.33	0.01 -0.26, 0.27
4	0.23 -0.07, 0.49	-0.19 -0.46, 0.12	-0.03 -0.29, 0.24	-0.08 -0.36, 0.22	-0.38 -0.61, -0.10	0.03 -0.26, 0.31	0.23 -0.05, 0.48	0.09 -0.20, 0.37	0.42 0.17, 0.63	0.37 0.08, 0.60	0.31 0.03, 0.55	0.35 0.05, 0.59	-0.04 -0.33, 0.24	0.27 -0.03, 0.53	0.40 0.14, 0.62	-0.00 -0.28, 0.28
5	0.16 -0.14, 0.44	-0.21 -0.47, 0.10	-0.11 -0.36, 0.17	-0.06 -0.35, 0.24	-0.38 -0.61, -0.10	0.00 -0.29, 0.30	0.14 -0.14, 0.41	0.09 -0.20, 0.37	0.41 0.15, 0.62	0.11 -0.19, 0.39	0.28 -0.01, 0.53	0.24 -0.07, 0.51	-0.18 -0.45, 0.12	0.22 -0.11, 0.50	0.30 0.01, 0.54	-0.09 -0.36, 0.20
6	0.21 -0.09, 0.47	0.06 -0.24, 0.35	-0.17 -0.42, 0.10	0.01 -0.28, 0.30	-0.14 -0.42, 0.15	0.09 -0.19, 0.36	0.23 -0.05, 0.48	0.18 -0.11, 0.44	0.52 0.30, 0.69	0.27 -0.01, 0.51	0.24 -0.04, 0.48	0.30 0.01, 0.54	0.13 -0.16, 0.38	0.22 -0.07, 0.47	0.09 -0.18, 0.35	-0.04 -0.30, 0.23
7	0.08 -0.22, 0.37	0.10 -0.20, 0.39	-0.01 -0.28, 0.26	-0.07 -0.35, 0.23	-0.29 -0.54, -0.00	-0.10 -0.37, 0.18	0.18 -0.11, 0.44	0.18 -0.11, 0.44	0.28 0.01, 0.52	0.34 0.06, 0.57	0.31 0.03, 0.54	0.39 0.10, 0.62	0.16 -0.14, 0.42	0.25 -0.05, 0.51	0.04 -0.24, 0.32	0.05 -0.23, 0.32
8	0.09 -0.21, 0.37	-0.07 -0.36, 0.24	-0.18 -0.43, 0.09	-0.18 -0.45, 0.12	-0.33 -0.57, -0.04	-0.12 -0.40, 0.19	0.21 -0.08, 0.46	0.05 -0.24, 0.34	0.47 0.22, 0.66	0.24 -0.06, 0.50	0.37 0.09, 0.59	0.34 0.04, 0.59	0.12 -0.18, 0.39	0.28 -0.04, 0.54	0.06 -0.22, 0.34	-0.15 -0.41, 0.14
9	-0.18 -0.42, 0.11	0.10 -0.18, 0.38	-0.02 -0.28, 0.24	-0.02 -0.30, 0.26	-0.15 -0.41, 0.13	-0.25 -0.49, 0.04	0.15 -0.12, 0.41	-0.23 -0.47, 0.06	0.45 0.20, 0.65	0.12 -0.18, 0.41	0.50 0.25, 0.69	0.22 -0.10, 0.50	-0.22 -0.49, 0.07	0.29 -0.03, 0.56	0.33 0.05, 0.57	0.14 -0.16, 0.41
10	0.29 -0.00, 0.53	0.02 -0.27, 0.32	-0.12 -0.37, 0.16	-0.22 -0.48, 0.08	-0.49 -0.68, -0.23	-0.01 -0.30, 0.29	0.15 -0.14, 0.41	0.12 -0.17, 0.39	0.49 0.27, 0.66	0.17 -0.11, 0.43	0.35 0.09, 0.56	0.19 -0.10, 0.45	-0.27 -0.50, -0.00	0.27 -0.03, 0.52	0.39 0.14, 0.59	0.04 -0.23, 0.30
11	0.10 -0.18, 0.37	0.01 -0.27, 0.30	-0.01 -0.27, 0.25	-0.19 -0.44, 0.11	-0.31 -0.55, -0.03	-0.07 -0.35, 0.23	0.12 -0.15, 0.39	-0.03 -0.31, 0.25	0.49 0.26, 0.67	0.31 0.02, 0.55	0.27 -0.00, 0.51	0.19 -0.12, 0.46	-0.17 -0.43, 0.11	0.33 0.01, 0.59	0.16 -0.11, 0.42	0.07 -0.20, 0.34

12	0.04 -0.23, 0.32	0.04 -0.25, 0.33	-0.00 -0.27, 0.26	-0.24 -0.49, 0.05	-0.28 -0.53, 0.01	-0.15 -0.41, 0.14	0.19 -0.09, 0.44	0.03 -0.26, 0.31	0.39 0.15, 0.58	0.30 0.03, 0.54	0.19 -0.08, 0.43	0.17 -0.12, 0.43	-0.22 -0.46, 0.05	0.28 -0.04, 0.54	0.24 -0.02, 0.47	0.03 -0.24, 0.29
13	0.27 -0.03, 0.52	-0.08 -0.36, 0.23	-0.26 -0.49, 0.02	-0.30 -0.54, 0.00	-0.36 -0.59, -0.07	-0.07 -0.34, 0.22	0.21 -0.08, 0.47	0.01 -0.28, 0.30	0.54 0.31, 0.71	0.22 -0.08, 0.49	0.42 0.14, 0.63	0.30 -0.01, 0.57	-0.12 -0.41, 0.17	0.37 0.07, 0.61	0.20 -0.08, 0.46	0.02 -0.26, 0.31
14	0.13 -0.16, 0.41	-0.11 -0.39, 0.19	-0.12 -0.37, 0.16	-0.12 -0.39, 0.18	-0.48 -0.68, -0.22	0.00 -0.29, 0.30	0.42 0.15, 0.63	-0.02 -0.31, 0.27	0.52 0.28, 0.69	0.22 -0.08, 0.49	-0.01 -0.31, 0.29	0.24 -0.08, 0.52	-0.15 -0.43, 0.14	0.29 -0.03, 0.55	0.42 0.13, 0.65	0.04 -0.24, 0.32
15	0.12 -0.17, 0.40	0.01 -0.28, 0.31	-0.05 -0.31, 0.23	-0.09 -0.37, 0.21	-0.32 -0.56, -0.03	-0.09 -0.37, 0.21	0.09 -0.20, 0.36	0.03 -0.26, 0.31	0.52 0.29, 0.70	0.22 -0.07, 0.49	0.46 0.19, 0.66	0.34 0.03, 0.59	-0.05 -0.34, 0.24	0.38 0.07, 0.62	0.28 -0.00, 0.52	0.11 -0.18, 0.38
16	0.00 -0.29, 0.30	0.16 -0.15, 0.44	-0.10 -0.36, 0.18	-0.31 -0.55, -0.01	-0.28 -0.53, 0.02	-0.16 -0.42, 0.13	-0.10 -0.37, 0.20	0.12 -0.17, 0.40	0.45 0.20, 0.64	0.17 -0.12, 0.44	0.10 -0.21, 0.38	0.37 0.07, 0.61	-0.13 -0.40, 0.16	0.39 0.10, 0.61	0.19 -0.11, 0.45	0.09 -0.19, 0.36
17	-0.01 -0.30, 0.29	-0.05 -0.34, 0.26	0.14 -0.13, 0.40	-0.11 -0.39, 0.20	-0.46 -0.66, -0.19	0.17 -0.12, 0.43	0.07 -0.22, 0.34	0.05 -0.24, 0.34	0.44 0.20, 0.63	0.04 -0.24, 0.33	-0.01 -0.28, 0.27	0.05 -0.25, 0.34	-0.17 -0.43, 0.12	0.09 -0.20, 0.37	0.29 0.01, 0.52	0.01 -0.26, 0.28
18	0.43 0.16, 0.64	0.19 -0.11, 0.46	-0.01 -0.28, 0.26	-0.42 -0.63, -0.14	-0.08 -0.37, 0.21	-0.36 -0.58, -0.09	0.08 -0.21, 0.35	0.02 -0.26, 0.30	0.18 -0.10, 0.44	0.18 -0.13, 0.45	0.32 0.03, 0.55	0.09 -0.23, 0.39	-0.12 -0.40, 0.18	0.11 -0.25, 0.43	0.08 -0.21, 0.35	-0.13 -0.39, 0.16
19	-0.21 -0.46, 0.09	-0.17 -0.43, 0.12	-0.19 -0.44, 0.08	-0.24 -0.50, 0.05	-0.31 -0.55, -0.03	-0.18 -0.43, 0.11	0.10 -0.18, 0.38	-0.13 -0.39, 0.16	0.30 0.06, 0.50	0.21 -0.09, 0.45	0.19 -0.06, 0.40	0.12 -0.16, 0.39	-0.19 -0.42, 0.07	0.10 -0.18, 0.35	0.29 0.04, 0.50	0.11 -0.18, 0.36
20	0.35 0.09, 0.57	-0.04 -0.32, 0.25	0.44 0.18, 0.64	0.10 -0.21, 0.39	-0.36 -0.59, -0.09	0.32 0.03, 0.56	0.25 -0.62, 0.52	0.18 -0.10, 0.43	0.21 -0.09, 0.48	0.20 -0.11, 0.47	-0.11 -0.37, 0.16	-0.25 -0.51, 0.05	-0.36 -0.59, -0.08	-0.15 -0.41, 0.14	-0.16 -0.46, 0.69	0.22 -0.07, 0.48
21	0.10 -0.21, 0.39	0.04 -0.27, 0.34	-0.18 -0.43, 0.10	-0.18 -0.45, 0.13	-0.43 -0.64, -0.14	-0.09 -0.36, 0.20	-0.15 -0.41, 0.15	-0.12 -0.40, 0.17	0.31 0.04, 0.54	-0.00 -0.29, 0.28	0.39 0.12, 0.60	0.03 -0.27, 0.33	-0.39 -0.61, -0.10	0.17 -0.13, 0.44)	0.18 -0.11, 0.43	0.18 -0.10, 0.43
22	0.46 0.21, 0.65	0.16 -0.13, 0.44	0.33 0.04, 0.56	0.35 0.02, 0.60	-0.01 -0.30, 0.27	0.32 0.02, 0.56	0.30 0.02, 0.54	0.03 -0.26, 0.31	0.10 -0.19, 0.38	-0.07 -0.35, 0.23	-0.07 -0.33, 0.21	-0.15 -0.42, 0.15	-0.50 -0.69, -0.24	-0.05 -0.32, 0.23	0.22 -0.06, 0.48	0.06 -0.23, 0.34
23	0.09 -0.20, 0.37	-0.13 -0.41, 0.18	-0.10 -0.34, 0.16	-0.13 -0.41, 0.17	-0.38 -0.61, -0.11	0.05 -0.23, 0.33	0.50 0.26, 0.68	-0.05 -0.32, 0.24	0.17 -0.10, 0.41	0.07 -0.21, 0.34	-0.36 -0.57, -0.10	-0.02 -0.30, 0.27	-0.17 -0.42, 0.10	0.03 -0.25, 0.30	0.15 -0.12, 0.40	-0.08 -0.33, 0.19

24	0.10 0.19, 0.39	-0.16 -0.43, 0.14	-0.09 -0.35, 0.18	-0.09 -0.37, 0.21	-0.43 -0.64, -0.16	0.05 -0.24, 0.32	0.52 0.28, 0.70	-0.06 -0.34, 0.23	0.46 0.22, 0.65	0.24 -0.05, 0.49	-0.07 -0.34, 0.22	0.21 -0.09, 0.48	-0.07 -0.35, 0.21	0.22 -0.08, 0.48	0.27 -0.00, 0.51	0.01 -0.27, 0.28
25	0.21 -0.09, 0.48	-0.10 -0.38, 0.20	-0.08 -0.34, 0.19	-0.08 -0.36, 0.22	-0.30 -0.55, -0.02	0.03 -0.25, 0.31	0.19 -0.10, 0.45	0.02 -0.27, 0.30	0.43 0.18, 0.64	0.28 -0.02, 0.53	0.43 0.16, 0.64	0.34 0.05, 0.58	-0.06 -0.35, 0.22	0.35 0.06, 0.58	0.23 -0.06, 0.48	-0.06 -0.33, 0.23
26	0.22 -0.07, 0.48	-0.07 -0.35, 0.23	-0.08 -0.34, 0.19	-0.11 -0.39, 0.19	-0.39 -0.61, -0.12	0.01 -0.27, 0.29	0.19 -0.09, 0.45	0.05 -0.24, 0.33	0.51 0.27, 0.69	0.21 -0.08, 0.48	0.46 0.20, 0.66	0.35 0.04, 0.59	-0.12 -0.40, 0.17	0.39 0.10, 0.62	0.29 0.01, 0.53	0.01 -0.27, 0.29
27	0.07 -0.22, 0.36	0.20 -0.11, 0.47	-0.01 -0.28, 0.27	-0.20 -0.46, 0.11	-0.42 -0.63, -0.14	-0.14 -0.42, 0.16	0.04 -0.25, 0.32	0.10 -0.19, 0.39	0.42 0.16, 0.62	0.02 -0.28, 0.33	0.28 -0.04, 0.53	0.20 -0.13, 0.49	-0.21 -0.48, 0.10	0.35 0.03, 0.62	0.26 -0.03, 0.51	0.05 -0.27, 0.34
28	0.28 -0.04, 0.56	-0.14 -0.41, 0.15	-0.10 -0.36, 0.19	-0.05 -0.34, 0.25	-0.30 -0.56, 0.00	0.02 -0.28, 0.31	0.18 -0.11, 0.44	0.06 -0.24, 0.36	0.43 0.16, 0.64	0.21 -0.07, 0.47	0.31 0.01, 0.56	0.31 0.01, 0.56	-0.10 -0.40, 0.21	0.28 -0.03, 0.54	0.25 -0.04, 0.49	-0.13 0.41, 0.17
29	-0.17 -0.42, 0.13	-0.00 -0.30, 0.31	-0.04 -0.30, 0.23	-0.30 -0.55, -0.01	-0.19 -0.45, 0.10	-0.42 -0.63, -0.14	0.25 -0.04, 0.49	-0.24 -0.50, 0.05	0.22 -0.05, 0.46	0.07 -0.25, 0.36	0.17 -0.11, 0.42	0.14 -0.16, 0.42	-0.07 -0.34, 0.20	0.12 -0.18, 0.40	0.27 -0.00, 0.51	-0.04 -0.30, 0.24
30	-0.02 -0.31, 0.27	0.10 -0.20, 0.38	0.06 -0.22, 0.32	0.02 -0.28, 0.31	0.41 0.13, 0.62	-0.06 -0.33, 0.23	-0.24 -0.49, 0.05	0.09 -0.21, 0.36	-0.35 -0.57, -0.08	-0.21 -0.48, 0.08	-0.24 -0.49, 0.05	-0.35 -0.59, -0.05	-0.14 -0.40, 0.15	-0.23 -0.49, 0.07	-0.30 -0.53, -0.02	0.11 -0.18, 0.37
31	0.14 -0.16, 0.42	-0.11 -0.39, 0.20	-0.09 -0.36, 0.20	-0.05 -0.34, 0.25	-0.30 -0.55, -0.01	0.03 -0.27, 0.33	0.29 0.00, 0.53	0.08 -0.21, 0.36	0.48 0.23, 0.67	0.28 -0.02, 0.53	0.28 -0.03, 0.54	0.35 0.04, 0.59	0.04 -0.25, 0.32	0.27 -0.04, 0.54	0.18 -0.11, 0.44	-0.07 -0.34, 0.21
32	0.00 -0.29, 0.30	0.13 -0.19, 0.42	-0.07 -0.34, 0.21	-0.41 -0.63, -0.13	-0.36 -0.59, -0.07	-0.32 -0.56, -0.02	-0.12 -0.39, 0.17	-0.07 -0.36, 0.22	0.37 0.10, 0.59	0.03 -0.27, 0.34	0.21 -0.10, 0.48	0.11 -0.22, 0.42	-0.36 -0.59, -0.07	0.31 -0.01, 0.59	0.33 0.05, 0.57	0.15 -0.14, 0.42

Cardiometabolic traits: A=systolicBP, B=diastolicBP, C=glucose, D=cholesterol, E=HDLcholesterol, F=LDLcholesterol, G=triglycerides, H=heart rate
Anthropometrics traits: 1=weight, 2=tricepsSF, 3=bicepsSF, 4=subscapularSF, 5=suprailiacSF, 6=calfSF, 7=frontthighSF, 8=abdominalSF, 9=calfC, 10=thighC, 11=upperarmC, 12=upperarmflexedC, 13=forearmC, 14=waistC, 15=hipC, 16=biacromialD, 17=bicristalD, 18=humerusD, 19=femurD, 20=height, 21=leglength, 22=bmi, 23=WHR, 24=WHtR, 25=BFmass, 26=BFfreemass, 27=percentageBF, 28=endomorphy, 29=mesomorphy, 30=ectomorphy, 31=factor1, 32=factor2
Abbreviations: BP=blood pressure, HDL=high-density lipoprotein, LDL=low-density lipoprotein, SF=skinfold, C=circumference, D=diameter, BF=body fat

Supplementary table S7. The predictive power of anthropometric indicators for borderline metabolic abnormalities in the pooled data of boys and girls.[1,2]

	Hypercholesterolemia 31%			Low HDL cholesterol 9%			High LDL cholesterol 14%			High triglycerides 28%			Hypertension 17%		
	AUC	95% CI		AUC	95% CI		AUC	95% CI		AUC	95% CI		AUC	95% CI	
		LL	UL		LL	UL		LL	UL		LL	UL		LL	UL
weight	0.54	0.48	0.60	0.58	0.49	0.68	0.62	0.55	0.69	0.62	0.56	0.68	0.62	0.56	0.68
tricepsSF	0.59	0.53	0.65	0.58	0.49	0.67	0.63	0.56	0.70	0.62	0.56	0.68	0.62	0.56	0.68
bicepsSF	0.59	0.53	0.65	0.58	0.48	0.67	0.64	0.56	0.71	0.62	0.56	0.68	0.62	0.56	0.68
subscapularSF	0.57	0.51	0.63	0.57	0.48	0.67	0.64	0.56	0.71	0.62	0.56	0.68	0.62	0.56	0.68
suprailiacSF	0.56	0.50	0.62	0.56	0.47	0.66	0.64	0.57	0.71	0.62	0.56	0.69	0.62	0.56	0.69
calfSF	0.58	0.52	0.64	0.57	0.47	0.67	0.63	0.56	0.70	0.62	0.56	0.68	0.62	0.56	0.68
frontthighSF	0.58	0.53	0.64	0.56	0.46	0.66	0.62	0.55	0.70	0.59	0.53	0.65	0.59	0.53	0.65
abdominalSF	0.57	0.51	0.63	0.57	0.47	0.66	0.63	0.56	0.70	0.62	0.56	0.68	0.62	0.56	0.68
calfC	0.58	0.52	0.64	0.57	0.47	0.67	0.63	0.56	0.70	0.62	0.56	0.68	0.62	0.56	0.68
thighC	0.54	0.48	0.60	0.58	0.48	0.67	0.60	0.53	0.68	0.61	0.55	0.67	0.61	0.55	0.67
upperarmC	0.54	0.48	0.60	0.59	0.50	0.68	0.62	0.55	0.69	0.63	0.57	0.69	0.63	0.57	0.69
upperarmflexedC	0.53	0.47	0.59	0.59	0.49	0.68	0.61	0.53	0.68	0.62	0.56	0.68	0.62	0.56	0.68
forearmC	0.54	0.48	0.60	0.59	0.50	0.68	0.61	0.54	0.68	0.61	0.55	0.67	0.61	0.55	0.67
waistC	0.56	0.50	0.62	0.60	0.51	0.69	0.63	0.56	0.70	0.65	0.59	0.71	0.65	0.59	0.71
hipC	0.55	0.49	0.61	0.59	0.49	0.68	0.60	0.53	0.67	0.60	0.54	0.66	0.60	0.54	0.66
biacromialD	0.59	0.53	0.66	0.57	0.47	0.66	0.59	0.51	0.66	0.58	0.52	0.64	0.58	0.52	0.64
bicristalD	0.58	0.51	0.64	0.57	0.48	0.66	0.59	0.51	0.66	0.58	0.52	0.64	0.58	0.52	0.64
humerusD	0.55	0.49	0.61	0.56	0.47	0.66	0.59	0.51	0.66	0.59	0.53	0.65	0.59	0.53	0.65
femurD	0.55	0.49	0.61	0.56	0.47	0.66	0.59	0.51	0.66	0.61	0.55	0.67	0.61	0.55	0.67
height	0.58	0.51	0.64	0.56	0.46	0.66	0.60	0.52	0.68	0.56	0.50	0.62	0.56	0.50	0.62
leglength	0.58	0.51	0.64	0.56	0.46	0.66	0.60	0.52	0.68	0.56	0.50	0.62	0.56	0.50	0.62
bmi	0.57	0.51	0.63	0.59	0.50	0.68	0.65	0.57	0.72	0.64	0.58	0.70	0.64	0.58	0.70
WHR	0.55	0.49	0.61	0.57	0.48	0.66	0.62	0.54	0.70	0.69	0.63	0.75	0.69	0.63	0.75
WHtR	0.59	0.53	0.65	0.62	0.53	0.71	0.66	0.59	0.74	0.68	0.62	0.73	0.68	0.62	0.73
BFmass	0.58	0.52	0.63	0.58	0.48	0.68	0.64	0.56	0.71	0.63	0.57	0.69	0.63	0.57	0.69
BFfreemass	0.57	0.51	0.63	0.57	0.48	0.66	0.59	0.52	0.66	0.60	0.54	0.66	0.60	0.54	0.66
percentageBF	0.58	0.52	0.64	0.58	0.49	0.67	0.63	0.56	0.70	0.63	0.57	0.69	0.63	0.57	0.69
endomorphy	0.58	0.52	0.64	0.58	0.48	0.67	0.64	0.57	0.71	0.63	0.57	0.69	0.63	0.57	0.69
mesomorphy	0.56	0.50	0.62	0.59	0.50	0.69	0.61	0.54	0.68	0.63	0.58	0.69	0.63	0.58	0.69
ectomorphy	0.59	0.53	0.65	0.60	0.51	0.69	0.65	0.58	0.73	0.65	0.59	0.70	0.65	0.59	0.70

[1]The models include age, the square of age, sex, and anthropometric indicators fitted individually in separate models.

[2]Limits used for the borderline metabolic abnormalities: ≥170 mg/dL for total cholesterol, ≥110 mg/dL for LDL cholesterol, ≤40 mg/dL for HDL cholesterol, ≥75 mg/dL in children younger than 10 years and ≥90 mg/dL for children 10 years of age and older for triglycerides, the highest decile of age adjusted SBP or DBP.

Abbreviations: AUC=the area under the receiver operating characteristic curve; CI=confidence interval, BP=blood pressure, HDL=high-density lipoprotein, LDL=low-density lipoprotein, SF=skinfold, C=circumference, D=diameter, BF=body fat
